# Association Between Obstructive Sleep Apnea and Brain White Matter Hyperintensities in a Population-Based Cohort in Germany

**DOI:** 10.1001/jamanetworkopen.2021.28225

**Published:** 2021-10-05

**Authors:** Helena U. Zacharias, Antoine Weihs, Mohamad Habes, Katharina Wittfeld, Stefan Frenzel, Tanweer Rashid, Beate Stubbe, Anne Obst, András Szentkirályi, Robin Bülow, Klaus Berger, Ingo Fietze, Thomas Penzel, Norbert Hosten, Ralf Ewert, Henry Völzke, Hans J. Grabe

**Affiliations:** 1 Department of Psychiatry and Psychotherapy, University Medicine Greifswald, Greifswald, Germany; 2 Department of Internal Medicine I, University Medical Center Schleswig-Holstein, Campus Kiel, Kiel, Germany; 3 Institute of Clinical Molecular Biology, Kiel University and University Medical Center Schleswig-Holstein, Campus Kiel, Kiel, Germany; 4 Neuroimage Analytics Laboratory and Biggs Institute Neuroimaging Core, Glenn Biggs Institute for Neurodegenerative Disorders, University of Texas Health Science Center at San Antonio, San Antonio; 5 Department of Radiology, University of Pennsylvania, Philadelphia; 6 German Centre for Neurodegenerative Diseases (DZNE), Site Rostock/Greifswald, Germany; 7 Department of Internal Medicine B–Cardiology, Pneumology, Infectious Diseases, Intensive Care Medicine, University Medicine Greifswald, Greifswald, Germany; 8 Institute of Epidemiology and Social Medicine, University of Muenster, Muenster, Germany; 9 Institute for Diagnostic Radiology and Neuroradiology, University Medicine Greifswald, Greifswald, Germany; 10 Interdisciplinary Centre of Sleep Medicine, University Hospital Charité Berlin, Berlin, Germany; 11 Institute for Community Medicine, Department Study of Health in Pomerania/Clinical Epidemiological Research, University Medicine Greifswald, Greifswald, Germany; 12 German Centre for Cardiovascular Research (DZHK), Partner Site Greifswald, Germany

## Abstract

**Importance:**

Underlying pathomechanisms of brain white matter hyperintensities (WMHs), commonly observed in older individuals and significantly associated with Alzheimer disease and brain aging, have not yet been fully elucidated. One potential contributing factor to WMH burden is chronic obstructive sleep apnea (OSA), a disorder highly prevalent in the general population with readily available treatment options.

**Objective:**

To investigate potential associations between OSA and WMH burden.

**Design, Setting, and Participants:**

Analyses were conducted in 529 study participants of the Study of Health in Pomerania–Trend baseline (SHIP-Trend-0) study with complete WMH, OSA, and important clinical data available. SHIP-Trend-0 is a general population–based, cross-sectional, observational study to facilitate the investigation of a large spectrum of common risk factors, subclinical disorders, and clinical diseases and their relationships among each other with patient recruitment from Western Pomerania, Germany, starting on September 1, 2008, with data collected until December 31, 2012. Data analysis was performed from February 1, 2019, to January 31, 2021.

**Exposures:**

The apnea-hypopnea index (AHI) and oxygen desaturation index (ODI) were assessed during a single-night, laboratory-based polysomnography measurement.

**Main Outcomes and Measures:**

The primary outcome was WMH data automatically segmented from 1.5-T magnetic resonance images.

**Results:**

Of 529 study participants (mean [SD] age, 52.15 [13.58] years; 282 female [53%]), a total of 209 (40%) or 102 (19%) individuals were diagnosed with OSA according to AHI or ODI criteria (mean [SD] AHI, 7.98 [12.55] events per hour; mean [SD] ODI, 3.75 [8.43] events per hour). Both AHI (β = 0.024; 95% CI, 0.011-0.037; *P *<.001) and ODI (β = 0.033; 95% CI, 0.014-0.051; *P *<. 001) were significantly associated with brain WMH volumes. These associations remained even in the presence of additional vascular, metabolic, and lifestyle WMH risk factors. Region-specific WMH analyses found the strongest associations between periventricular frontal WMH volumes and both AHI (β = 0.0275; 95% CI, 0.013-0.042, *P* < .001) and ODI (β = 0.0381; 95% CI, 0.016-0.060, *P* < .001) as well as periventricular dorsal WMH volumes and AHI (β = 0.0165; 95% CI, 0.004-0.029, *P* = .008).

**Conclusions and Relevance:**

This study found significant associations between OSA and brain WMHs, indicating a novel, potentially treatable WMH pathomechanism.

## Key Points

**Question:**

Is obstructive sleep apnea (OSA) associated with brain white matter hyperintensities (WMHs)?

**Findings:**

In this cohort study of 529 participants of the Study of Health in Pomerania-Trend baseline, a statistically significant association was found between increased OSA and increased brain WMHs.

**Meaning:**

The associations found in this study between OSA and brain WMHs may indicate a novel, potentially treatable white matter disease pathomechanism.

## Teaser

This cohort study assesses whether the increased burden of obstructive sleep apnea is associated with white matter hyperintensity load and investigates the influence of additional metabolic, vascular, and lifestyle white matter hyperintensity risk factors on this association.

## Introduction

Brain white matter hyperintensities (WMHs) are commonly observed on magnetic resonance imaging (MRI) of older individuals, those with dementia, and patients with stroke.^1,2,3,4,5^ Brain WMHs are typically suggested to be a marker of cerebral small vessel disease, alongside other lesions, such as lacunar infarctions or microbleeds.^1,2,4^ White matter hyperintensities are associated with an increased risk of dementia, cognitive decline, stroke, death, abnormal gait, disturbed balance, and depression.^1,2,4,6,7^ Habes et al^5,8^ reported higher WMH burden to be associated with advanced brain aging and increased brain atrophy patterns related to Alzheimer disease (AD) in the Study of Health in Pomerania (SHIP). A recent longitudinal study^9^ reported associations between greater WMH burden and accelerated cognitive, neuropsychiatric, and functional decline independent of traditional AD risk factors and MRI biomarkers.

Vascular risk factors, including hypertension, diabetes, and smoking, but also age, male sex, increasing systolic blood pressure, and lower educational level have been associated with increased WMH values.^1,3,4,5^ Brain pathological analysis found focal myelinolysis, axonal loss, and gliosis associated with vessel wall hyalinosis in regions with white matter disease.^4,5,10^ Potential pathogenic mechanisms of WMH appearance could include ischemia or hypoxia, hypoperfusion attributable to altered cerebrovascular autoregulation, inflammation, and subsequent demyelination.^2,3,4^ Nevertheless, our knowledge regarding WMH pathophysiology remains limited because of small-scale studies^1,3^ in specific subpopulations. Consequently, the search for preventive and therapeutic strategies to reduce WMH burden is ongoing.^3^

Ischemia or hypoxia and hypoperfusion are main characteristics of obstructive sleep apnea (OSA), a common manifestation of chronic sleep-disordered breathing. This condition is caused by a recurrent upper-airway collapse during sleep, leading to brain arousal, sympathetic activation, and blood oxygen desaturation.^11^ The diagnosis of OSA is most reliably performed by overnight polysomnography (PSG) because this disorder often lacks symptoms.^11,12,13^ Typically, OSA severity is categorized by the apnea-hypopnea index (AHI), a combined measure of airflow absence or reduction accompanied by oxygen desaturations or arousals. The oxygen desaturation index (ODI),^13,14^ which focuses on oxygen level decreases, appears to be of similar importance and can be assessed by a cheaper and less burdensome pulse oximetry measurement.^15,16^ The prevalence of OSA is highly diverse in the general population, ranging from 9% to 38%, with older age, male sex, and obesity as known risk factors.^11,13,17^ In advanced age groups, the prevalence can increase to 84%.^11^ Obstructive sleep apnea has been identified as a significant risk factor for cardiovascular, metabolic, and psychiatric disorders.^11,13^ So far, however, the association between OSA and white matter disease, both highly prevalent in older individuals, has been investigated by only a few human studies,^10,18,19,20,21^ with contradictory findings. Previous studies suffer from restrictions to specific subpopulations^10^ with high comorbidity burden^20,21^ or because they used nonuniform OSA assessment methods,^10^ were carried out in an in-home setting,^19,20,21^ were based on sleep questionnaire data only,^22^ or had relatively small sample sizes,^20^ and few used fully automated WMH ratings.^22^ Thus, additional general population studies that investigate the association between OSA and WMHs with highly standardized data collection and thorough covariate adjustment are needed. With OSA treatment options (eg, positive airway pressure therapy) readily available,^18^ WMHs and associated diseases, including subsequent dementia, might be reduced.

SHIP-Trend offers a large-scale, general population study sample with highly standardized PSG and MRI-based WMH data. We hypothesized that the increased burden of OSA is positively associated with WMH load and investigated the influence of additional metabolic, vascular, and lifestyle WMH risk factors on this association. We further tested possible 2-way interactions between OSA and these risk factors and specific OSA associations in individual brain WMH regions.

## Methods

### Study Population

We included 529 study participants from the SHIP-Trend baseline (SHIP-Trend-0) study, a general population–based, cohort study, who were randomly recruited from the adult population in Western Pomerania, Germany, in September 2008 and led by the Institute for Community Medicine, University Medicine Greifswald.^23^ The ethics committee of the University Medicine Greifswald approved the SHIP study and this analysis, and written informed consent was provided by all study participants. The original SHIP-Trend-0 cohort included 4420 participants, and PSG data could be obtained from a subset of 1109. For 607 of these 1109 study participants, WMH and intracranial volume (ICV) data were available. After the exclusion of another 78 individuals because of missing demographic and clinical chemistry data as well as after normality assessment of the regression residuals, the final study sample included 529 individuals. All data were deidentified. This study follows Strengthening the Reporting of Observational Studies in Epidemiology (STROBE) reporting guideline.

### Data Assessment

Clinical examinations by computer-assisted face-to-face interviews and subsequent medical examinations were performed from January 1, 2008, to December 31, 2012 (eMaterials and eMethods in the Supplement). Data analysis was performed from February 1, 2019, to January 31, 2021. Each SHIP-Trend-0 study participant was offered a 1-night PSG session, including a sleep questionnaire and a whole-body MRI measurement, unless any contraindications were present.

### MRI Acquisition and Preprocessing

T1-weighted and fluid-attenuated inversion recovery MRIs were used for WMH determination. Image acquisition on a 1.5-T MRI scanner (Magnetom Avanto, Siemens Medical Systems) has been previously detailed^24^ (eMaterials and eMethods in the Supplement).

Preprocessing of the MRI data is detailed in Habes et al.^5^ In brief, extracranial material was removed (skull stripping) by a multi–Atlas-based algorithm,^25^ followed by visual inspection for quality control. Images were corrected for bias field,^26^ and tissue segmentation into gray matter, white matter, and cerebrospinal fluid was performed with an in-house algorithm.^27^ The ICV was calculated using an individual’s binary brain mask and defined as the total of white matter, gray matter, and cerebrospinal fluid.

### WMH Segmentation

White matter hyperintensity segmentation was fully automated according to Habes et al.^5^ After the coregistration of fluid-attenuated inversion recovery and T1-weighted images to the same space, WMHs were segmented with a support vector machine–based method and visually inspected for quality control. The minimum WMH volume was set to 3 mm^3^. Then WMH total volumes and the number of WMH spots with a volume larger than 3 mm^3^, hereafter referred to as WMH counts, were determined.^28^ The WMH volumes within 4 specific brain regions were determined by a nonnegative matrix factorization method, which summarizes complex multivariate covariation patterns with a predefined number of components.^29^ These 4 components were specified as periventricular posterior, periventricular frontal, periventricular dorsal, and deep white matter regions and were previously reported to have differential associations with vascular and AD risk factors.^29^

### OSA Parameter Assessment

Study participants attended a single-night, laboratory-based PSG (Alice 5 System, Philips Respironics) at a study site in Greifswald, Germany,^30^ a mean of 9 days after the baseline examinations.^13^ Sleep and breathing events were visually scored according to the American Academy of Sleep Medicine 2012 criteria.^31^ An apnea event was scored if a decrease in air flow peak signal excursion by 90% or greater of preevent baseline for at least 10 seconds occurred. A hypopnea event was scored if a flow decrease of 30% or greater of preevent baseline for at least 10 seconds associated with a 3% or greater oxygen desaturation or an arousal occurred. The AHI was defined as the number of apnea and hypopnea events per hour of total sleep time. The ODI was defined as the number of 4% or greater oxygen desaturations per hour of total sleep time determined by pulse oximetry (eMaterials and eMethods in the Supplement).

### Statistical Analysis

Statistical analyses were performed with *R* software, version 3.6.0,^32^ using the R packages tidyverse,^33^ ggplot2,^34^ and ggpubr^35^ for data visualization (R Foundation for Statistical Computing). For descriptive analyses, groups were compared with the Pearson χ^2^ tests for categorical variables and 2-sided *t* tests, assuming unequal variance for continuous variables.

Possible associations between WMHs (outcome) and OSA variables (explanatory variables) were tested by linear multivariable regression analyses. The WMH volume and count data were log_2_ transformed after the addition of a pseudo-count of 1 to normalize their distributions. The null model comprised age modeled by a restricted cubic spline with 4 knots located at the 5%, 33%, 66%, and 95% quantiles, using the R package rms,^36^ sex, ICV, and body height as covariates. Significant associations between age and the respective outcome variable were tested by Wald tests with the R package car.^37^ The OSA model comprised, in addition to all null model covariates, the respective OSA variable. In subsequent sensitivity analyses, OSA models were extended by additional vascular, metabolic, and lifestyle WMH risk factors. Furthermore, 2-way interactions between AHI or ODI and the aforementioned additional risk factors were tested. A 2-sided *P* < .05 was considered to be statistically significant.

Association and sensitivity analyses were performed again with the 4 region-specific WMH volume compartments available for a subsample of 392 SHIP-Trend-0 study participants (eFigure 1 in the Supplement). We accounted for multiple testing according to Bonferroni, and the statistical significance threshold was set to .0125 (.05 / 4).

## Results

### Baseline Characteristics of the Study Sample

Of 529 study participants (mean [SD] age, 52.15 [13.58] years; 282 female [53%]), a total of 209 (40%) or 102 (19%) individuals were diagnosed with OSA according to AHI or ODI criteria (Table 1). In general, the included compared with the excluded SHIP-Trend-0 sample exhibited more favorable health parameters, had higher educational levels, and reported more sleep problems (eTable 1 in the Supplement).

**Table 1.** Characteristics of SHIP-Trend-0 Sample Included in this Study^a^

Characteristic	SHIP-Trend-0 subcohort finding (n = 529)
Age, mean (SD), y	52.2 (13.6)
Systolic blood pressure, mean (SD), mm Hg	126.83 (16.98)
Diastolic blood pressure, mean (SD), mm Hg	77.36 (9.44)
Glycated hemoglobin, mean (SD), %	5.33 (0.73)
Total cholesterol, mean (SD), mg/dL	214.67 (42.08)
Total triglycerides, mean (SD), mg/dL	139.82 (99.12)
HDL-C, mean (SD), mg/dL	55.60 (13.51)
LDL-C, mean (SD), mg/dL	134.36 (35.53)
Waist circumference, mean (SD), cm	90.16 (12.78)
Hip circumference, mean (SD), cm	102.28 (9.17)
Height, mean (SD), cm	169.87 (9.08)
Weight, mean (SD), kg	80.90 (14.62)
BMI, mean (SD)	27.99 (4.34)
C-reactive protein, mean (SD), mg/dL	0.26 (0.51)
Fibrinogen, mean (SD), mg/dL	308 (74)
White blood cells, /μL	5810 (2710)
Alcohol consumption within last 30 d, mean (SD), g/d	8.65 (11.88)
Subjective mental health summary scale score, mean (SD)	52.98 (8.50)
Diabetes (type 1 or type 2)	53 (10)
Sex, women	282 (53)
Cigarette smoking	
Never-smoker	233 (44)
Ex-smoker	202 (38)
Current smoker	94 (18)
Physically active, No. (%)	392 (74)
Educational level, y	
<10	71 (13)
10	278 (53)
>10	180 (34)
Lifetime depression	163 (31)
Medication	
Antidiabetic drugs	25 (5)
Antihypertensive drugs	184 (35)
Lipid-lowering drugs	48 (9)
Hypertension	240 (45)
Sleep time normal workday, mean (SD), h	6.92 (1.26)
No nap in last 7 d	307 (58)
How often, within 1 week, does it take >30 min for the individual to fall asleep?	
4-7	104 (20)
1-3	116 (22)
<1	84 (16)
0	224 (42)
Don’t know	1 (0)
No. of times awake during night for >30 min per week	
4-7	64 (12)
1-3	81 (15)
<1	83 (16)
0	134 (25)
Often wakes up during night but gets back to sleep	166 (31)
Don’t know	1 (0)
Answer refused	0
Snoring	
Regularly	141 (27)
Occasionally	245 (46)
Never	106 (20)
Don’t know	37 (7)
Magnetic resonance imaging parameters	
Intracranial volume, mean (SD), mm^3^	1.58 × 10^6^ (0.16 × 10^6^)
WMH volume, median (IQR), mm^3^	208 (83-590)
WMH counts, mean (SD)	13.55 (10.61)
Polysomnography parameters	
Total sleep time, mean (SD), h	6.19 (1.06)
Wake after sleep onset, mean (SD), min	62.16 (44.60)
Sleep efficiency, %	81.13 (11.84)
Time in sleep stage, % per TST	
REM	18.34 (5.90)
N1	14.56 (8.94)
N2	52.58 (7.80)
N3	14.51 (8.17)
AHI, mean (SD), events per hour of TST	7.98 (12.55)
AHI categories	
No sleep apnea (AHI <5 per hour of TST)	320 (60)
Mild sleep apnea (AHI 5-15 per hour of TST)	125 (24)
Moderate sleep apnea (AHI 15-30 per hour of TST)	52 (10)
Severe sleep apnea (AHI ≥30 per hour of TST)	32 (6)
ODI, mean (SD), events per hour of TST	3.75 (8.43)
ODI categories	No. (%)
No sleep apnea (ODI <5 per hour of TST)	427 (81)
Mild sleep apnea (ODI 5-15 per hour of TST)	69 (13)
Moderate sleep apnea (ODI 15-30 per hour of TST)	20 (4)
Severe sleep apnea (ODI ≥30 per hour of TST)	13 (2)

Abbreviations: AHI, apnea hypopnea index; BMI, body mass index (calculated as weight in kilograms divided by height in meters squared); HDL-C, high-density lipoprotein cholesterol; LDL-C, low-density lipoprotein cholesterol; ODI, oxygen desaturation index; TST, total sleep time.

SI conversion factors: To convert hemoglobin to proportion of total hemoglobin, multiply by 0.01; total cholesterol, HDL-C, and LDL-C to millimoles per liter, multiply by 0.0259; triglycerides to millimoles per liter, multiply by 0.0113; C-reactive protein to milligrams per liter, multiply by 10; fibrinogen to grams per liter, multiply by 0.01; and white blood cells to ×10^9^/L, multiply by 0.001.

^a^ Data are presented as number (percentage) of patients unless otherwise indicated.

### Prevalence of OSA in the Study Sample

A total of 209 individuals (40%) were diagnosed with OSA according to AHI criteria, with a mean (SD) AHI of 7.98 (12.55) events per hour in the complete sample (Table 1). Within this OSA group, 125 (24% of the complete study sample) had mild, 52 (10%) had moderate, and 32 (6%) had severe OSA. According to ODI criteria, a total of 102 individuals (19%) were diagnosed with OSA, of whom 69 (13%) had mild, 20 (4%) had moderate, and 13 (2%) had severe OSA. The mean (SD) ODI in the complete study sample amounted to 3.75 (8.43) events per hour.

Individuals with compared with those without OSA were older (mean [SD] age: AHI criteria: 58.6 [10.3] vs 47.9 [13.8] years; ODI criteria: 60.5 [10.3] vs 50.2 [13.5] years), had higher blood pressures (mean [SD] systolic blood pressure: AHI criteria: 133.17 [16.96] vs 122.69 [15.68] mm Hg; ODI criteria: 135.98 [17.39] vs 124.64 [16.14] mm Hg; mean [SD] diastolic blood pressure: AHI criteria: 79.98 [10.18] vs 75.64 [8.52] mm Hg; ODI criteria: 81.80 [11.18] vs 76.29 [8.66] mm Hg), and higher glycated hemoglobin values (mean [SD]: AHI criteria: 5.59% [0.79%] vs 5.17% [0.63%]; ODI criteria: 5.71% [0.84%] vs 5.24% [0.67%] [to convert to proportion of total hemoglobin, multiply by 0.01]) and, consequently, more often had diabetes (AHI criteria: 37 [18%] vs 16 [5%]; ODI criteria: 23 [23%] vs 30 [7%]), higher fibrinogen (mean [SD]: AHI criteria: 325 [83] vs 297 [65] mg/dL; ODI criteria: 326 [79] vs 304 [72] mg/dL [to convert to grams per liter, multiply by 0.01]), triglyceride (mean [SD]: AHI criteria: 168.14 [118.58] vs 122.12 [78.76] mg/dL; ODI criteria: 170.80 [132.74] vs 132.74 [87.61] mg/dL [to convert to millimoles per liter, multiply by 0.0113]), and low-density lipoprotein cholesterol (LDL-C) levels (mean [SD]: AHI criteria: mean [SD] 140.15 [33.20] vs 130.50 [36.30] mg/dL; ODI criteria: 140.93 [31.66] vs 132.82 [36.29] mg/dL [to convert to millimoles per liter, multiply by 0.0259]) but lower high-density lipoprotein cholesterol (HDL-C) levels (mean [SD]: AHI criteria: 51.35 [11.20] vs 58.69 [14.29] mg/dL; ODI criteria: 50.58 [11.58] vs 56.76 [13.90] mg/dL [to convert to millimoles per liter, multiply by 0.0259]), and higher anthropomorphic measures (mean [SD] waist circumference: AHI criteria: 96.76 [11.36] vs 85.85 [11.80] cm; ODI criteria: 100.46 [11.51] vs 87.70 [11.82] cm; hip circumference: AHI criteria: 105.04 [8.83] vs 100.48 [8.94] cm; ODI criteria: 106.73 [8.52] vs 101.22 [9.01] cm; body weight: AHI criteria: 86.54 [14.13] vs 77.21 [13.76] kg; ODI criteria: 90.21 [14.72] vs 78.68 [13.71] kg; body mass index [BMI; calculated as weight in kilograms divided by height in meters squared]: AHI criteria: 29.91 [4.10] vs 26.74 [4.03]; ODI criteria: 31.04 [4.03] vs 27.26 [4.09]; mean [SD] subjective mental health summary scale score: AHI criteria: 53.67 [8.66] vs 52.52 [8.37]; ODI criteria: 53.46 [8.60] vs 52.86 [8.28]). They also had lower educational levels (<10 years: AHI criteria: 40 [19%] vs 31 [10%]; ODI criteria: 25 [25%] vs 46 [11%]), took more medications (antihypertensive drug use: AHI criteria: 105 [50%] vs 79 [25%]; ODI criteria: 59 [58%] vs 125 [29%]), more frequently had hypertension (AHI criteria: 132 [63%] vs 108 [34%]; ODI criteria: 75 [74%] vs 165 [39%]), snored more (regular snoring: AHI criteria: 84 [40%] vs 57 [18%]; ODI criteria: 49 [48%] vs 92 [22%]), and took more naps during the daytime (no nap in last 7 days: AHI criteria: 94 [45%] vs 213 [67%]; ODI criteria: 40 [39%] vs 267 [63%]) (eTable 2 in the Supplement). Significantly more men than women (AHI criteria: 199 [62%] women without OSA vs 83 [40%] women with OSA; *P* < .001; ODI criteria: 248 [58%] women without OSA vs 34 [33%] women with OSA; *P* < .001) and more ex-smokers (AHI criteria: 103 [32%] vs 99 [47%]; ODI criteria: 149 [35%] ex-smokers without OSA vs 53 [52%] ex-smokers with OSA; *P* < .001) were diagnosed with OSA.

### Association of OSA With Brain WMHs

The WMH volumes were significantly, positively associated with both AHI (β = 0.024; 95% CI, 0.011-0.037; *P *<.001) and ODI (β = 0.033; 95% CI, 0.014-0.051; *P *<.001) in multivariable linear regression models adjusted for sex, age, ICV, and body height (Table 2). Likewise, WMH counts were significantly, positively associated with both AHI (β = 0.008; 95% CI, 0.002-0.014; *P* = .01) and ODI (β = 0.011; 95% CI, 0.0018-0.020; *P* = .02) (eTable 3 in the Supplement). An increasing effect size for increasing OSA severity was observed (eTable 4 in the Supplement).

**Table 2.** Results of the Linear Regression Analysis for WMH Volumes With Respect to Obstructive Sleep Apnea Diagnostic Criteria

Variable	WMH volume^a^					
	Null model^b^			AHI or ODI model^b^		
	β (SE)	*P* value^c^		β (SE)	*P* value^c^	
**AHI regression models**						
Constant	–0.581 (2.438)	.81		–0.650 (2.409)	.79	
AHI	NA	NA		0.024 (0.006)	<.001	
Female sex	–0.232 (0.227)	.31		–0.064 (0.229)	.78	
rcs age	0.089 (0.021)	<.001	<.001	0.083 (0.021)	<.001	<.001
rcs age′	0.031 (0.045)	.50		0.029 (0.045)	.53	
rcs age′′	–0.112 (0.225)	.62		–0.104 (0.223)	.64	
ICV	3 × 10^−6^ (1 × 10^−6^)	<.001		4 × 10^−6^ (1 × 10^−6^)	<.001	
Height	–0.011 (0.013)	.38		–0.012 (0.013)	.37	
Observations	529					
*R^2^*	0.425			0.439		
Adjusted *R^2^*	0.418			0.432		
Residual SE	1.744 (*df* = 522)			1.723 (*df* = 521)		
**ODI regression models**						
Constant	–0.581 (2.438)	.81		–1.015 (2.416)	.68	
ODI	NA	NA		0.033 (0.009)	.001	
Female sex	–0.232 (0.227)	.31		–0.079 (0.229)	.73	
rcs age	0.089 (0.021)	<.001	<.001	0.086 (0.021)	<.001	<.001
rcs age′	0.031 (0.045)	.50		0.029 (0.045)	.52	
rcs age′′	–0.112 (0.225)	.62		–0.110 (0.223)	.62	
ICV	3 × 10^−6^ (1 × 10^−6^)	<.001		4 × 10^−6^ (1 × 10^−6^)	<.001	
Height	–0.011 (0.013)	.38		–0.010 (0.013)	.44	
Observations	529					
*R^2^*	0.425			0.438		
Adjusted *R^2^*	0.418			0.430		
Residual SE	1.744 (*df* = 522)			1.726 (*df* = 521)		

Abbreviations: AHI, apnea-hypopnea index; ICV, intracranial volume; NA, not applicable; ODI, oxygen desaturation index; rcs, restricted cubic spline with 4 knots located at the 5%, 33%, 66%, and 95% quantiles, with rcs age, rcs age′, and rcs age′′ representing the respective coefficients of the restricted cubic spline regression; WMH, white matter hyperintensity.

^a^ The WMH volume data have been log_2_ transformed after the addition of a pseudocount of 1.

^b^ The null model regressed the independent variable WMH volumes on the explanatory variables sex, age modeled by an rcs, intracranial volume, and height. The OSA model extended the explanatory variables of the null model by AHI or ODI, respectively.

^c^ *P* values were calculated using the Wald test.

**Figure 1.**
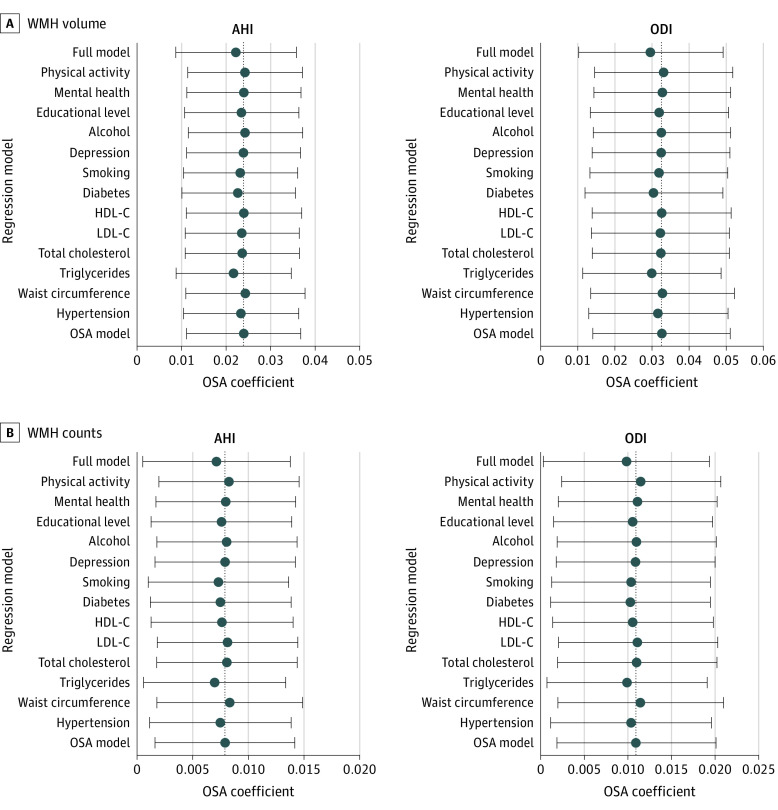
Estimated Effect Sizes of Obstructive Sleep Apnea (OSA) Parameters on White Matter Hyperintensities (WMHs) in Extended Regression Models The x-axis gives the estimated effect sizes (regression coefficients β) and 95% CIs of OSA on WMH volumes and counts. Obstructive sleep apnea was defined by the apnea-hypopnea index (AHI) or the oxygen desaturation index (ODI). The OSA regression models were extended 1 by 1 by the additional confounder variables given on the y-axis. The dashed vertical lines mark the respective OSA β-coefficients in the OSA models only adjusted for sex, age modeled by a restricted cubic spline, intracranial volume, and body height. The full model includes the respective OSA parameter, the complete set of metabolic, vascular, and lifestyle covariates, as well as the null model covariates. HDL-C indicates high-density lipoprotein; LDL-C, low-density lipoprotein cholesterol.

Both AHI and ODI had the strongest associations with WMHs compared with other metabolic, vascular, and lifestyle WMH risk factors, followed by diabetes, triglyceride levels, and smoking (eFigure 2 in the Supplement). After the OSA models were adjusted for these risk factors in a 1-by-1 fashion and for all confounders simultaneously, the continuous OSA parameters were still strongly associated with WMHs independent of established WMH risk factors (Figure 1 and eTable 5 in the Supplement). Similar results were obtained for categorized OSA variables (eFigure 3 and eTable 6 in the Supplement).

### Two-Way Interactions Between OSA and Metabolic, Vascular, and Lifestyle Risk Factors on WMHs

We explored 2-way interactions between continuous AHI and ODI parameters and the following vascular, metabolic, and lifestyle WMH risk factors: hypertension, waist circumference, triglyceride, total cholesterol, LDL-C and HDL-C levels, diabetes, lifetime depression, subjective mental health, smoking, daily alcohol consumption, educational level, and physical activity on WMH volumes and counts (eTable 7 in the Supplement). No significant interactions could be observed for AHI, whereas for ODI, 1 significant interaction (β = 0.030; 95% CI, 0.0039-0.056, *P* = .03) with current smoking on WMH counts was identified.

### Specific Associations of OSA With Individual Brain WMH Regions

**Figure 2.**
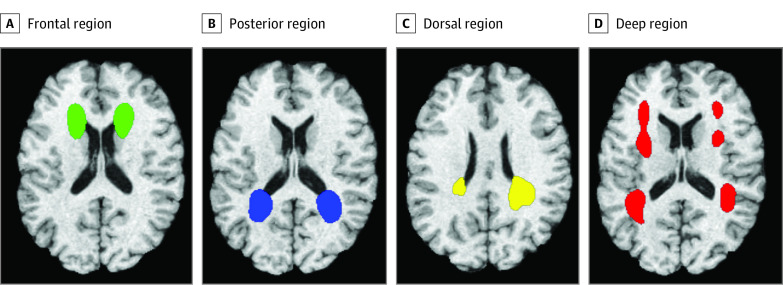
White Matter Hyperintensities Decomposed in 4 Regional Patterns as Described by Habes et al^29^ These areas delineated as described by Habes et al.^29^ Frontal, posterior, dorsal, and deep regions are plotted in green, blue, yellow, and red, respectively.

Association analyses were repeated in 4 specific WMH regions (Figure 2). All region-specific WMH volumes showed positive associations with both continuous OSA parameters, with strongest, statistically significant associations between periventricular frontal WMH volumes and both AHI (β = 0.0275; 95% CI, 0.013-0.042; *P *<.001) and ODI (β = 0.0381; 95% CI, 0.016-0.060; *P *<.001), as well as periventricular dorsal WMH volumes and AHI (β = 0.0165; 95% CI, 0.004-0.029; *P* = .008) (Table 3). After adjustment for additional metabolic, vascular, and lifestyle risk factors, AHI or ODI parameters were still strongly associated with periventricular frontal WMH volumes (eTable 8 in the Supplement). Associations between periventricular dorsal WMH volumes and AHI were no longer significant after adjustment for triglyceride (β = 0.013; *P* = .03) and HDL-C (β = 0.015; *P* = .02) levels, diabetes (β = 0.015; *P* = .02), physical activity (β = 0.015; *P* = .02), and all additional covariates simultaneously (β = 0.013; *P* = .05).

**Table 3.** Specific Obstructive Sleep Apnea Associations With WMH Volumes in Individual Brain Regions Adjusted for Sex, Age Modeled by a Restricted Cubic Spline, Intracranial Volume, and Height in a Subsample of 392 SHIP-Trend-0 Study Participants

Variable^a^	Apnea-hypopnea index regression models		Oxygen desaturation index regression models	
	β (SE)	*P* value	β (SE)	*P* value
Periventricular frontal WMH volume	0.0275 (0.0075)	<.001	0.0381 (0.0112)	<.001
Periventricular dorsal WMH volume	0.0165 (0.0062)	.008	0.0171 (0.0093)	.07
Periventricular posterior WMH volume	0.0145 (0.0078)	.07	0.0118 (0.0117)	.32
Deep white matter WMH volume	0.0085 (0.0059)	.15	0.0053 (0.0088)	.55

Abbreviations: SHIP-Trend-0, Study of Health in Pomerania–Trend baseline; WMH, white matter hyperintensity.

^a^ Regional WMH volumes have been log_2_ transformed after addition of a pseudocount of 1.

## Discussion

This cohort study found positive, statistically significant associations between brain WMHs and OSA, represented by the AHI and ODI, in the general population. These results might indicate a novel option to reduce WMH burden via this modifiable risk factor. Associations between WMH and OSA strongly depended on OSA severity, suggesting a dose-dependent association. The associations remained significant after thorough adjustment for additional metabolic, vascular, and lifestyle WMH risk factors, indicating a strong, independent link between OSA and WMH formation. Of interest, we observed brain region–specific associations between WMHs and OSA, with the strongest associations in periventricular frontal and dorsal WMH compartments.

Recurrent altered blood oxygen supply and blood pressure attributable to apnea or hypopnea events may cause ischemia or hypoxia and hypoperfusion of brain white matter tissue, potentially representing a major pathomechanism of WMH formation.^10^ Inflammation might be another pathomechanism; however, in our study, we could not detect any significant causal mediation of OSA associations with WMHs by common inflammation markers, including C-reactive protein, white blood cell count, and fibrinogen. In contrast, Weihs et al^38^ reported a significant causal mediation effect of white blood cell count on the association between OSA and brain age. This finding possibly indicates different pathomechanisms of OSA associations with brain WMH formation compared with brain age.

Habes et al^29^ reported, on the basis of a subsample of the SHIP-Trend-0 cohort, significant associations between periventricular dorsal WMHs and AD genetic risk as well as longitudinal cognitive decline.^29^ Furthermore, they found strong associations between periventricular frontal WMH compartments, which also appeared earlier in life than the other 3 WMH compartments, and blood pressure as well as cortical atrophy.^29^ Thus, one might speculate that these 2 specific WMH compartments, which seem to be more strongly associated with OSA and blood pressure alterations and more strongly associated with AD genetic risk than the other 2 WMH compartments, might be the most interesting target regions to investigate causal relationships among OSA, WMHs, and AD in future studies. However, associations between periventricular dorsal WMHs and OSA vanished in our study after adjustment for triglyceride and HDL-C levels, diabetes, and physical activity. Thus, thorough consideration of these confounders in future studies seems to be necessary. Another study^39^ found increased tau positron emission tomography levels in the entorhinal and inferior temporal cortices in cognitively unimpaired older individuals compared with those without witnessed sleep apneas. This finding might indeed point to an increased susceptibility to tau accumulation and thus increased risk of AD in individuals with OSA.^39^

Positive associations between WMHs and OSA diagnosed by overnight PSG have also been reported in a Korean general population study.^10^ Again, OSA associations with WMH presence increased with increasing OSA severity in a dose-dependent manner and remained statistically significant after adjustment for hypertension.^10^ In contrast, Lutsey et al^19^ reported no statistically significant association of OSA with WMH volumes in 312 Atherosclerosis Risk in Communities study participants. Potential reasons for these different findings compared with our study might be the investigation of a smaller and slightly older study sample with PSG measurements only available from less reliable portable devices performed in an unattended, in-home setting and a rather large gap of 15 years between OSA and MRI measurements in the Atherosclerosis Risk in Communities study.^19^ Likewise, the lack of statistically significant associations of OSA with global WMHs in 28 patients with heart failure and 109 patients with a minor stroke or transient ischemic attack after adjustment for age, hypertension, and diabetes might have been attributable to the rather small study samples and in-home PSG measurements.^20,21^

### Strengths and Limitations

This study has several strengths. To our knowledge, it included one of the largest, general population samples with highly standardized MRI, PSG, and confounder data studied to date. The SHIP-Trend-0 WMH data were generated by an automatic, machine learning–based segmentation procedure providing quantitative and objective WMH measures, and PSG data measurements and abstraction were performed by trained personnel according to well-defined criteria.

This study also has limitations. No causal conclusions can be drawn from our results because they are derived from cross-sectional data only. Future studies should elucidate potential causal relationships between OSA and WMH formation in the general population. Remarkably, no clear interactions between AHI or ODI and other WMH risk factors could be established. Putative interactions with alcohol consumption, physical activity, diabetes, LDL-C levels, and smoking should be reassessed in future studies. SHIP-Trend-0 only included individuals of European White ancestry, and OSA associations with WMH might differ in other populations. Likewise, we could only include 12% of the original cohort into our study, which might have resulted in a selection bias that influenced our findings because the included compared with the excluded study sample was slightly healthier, was more educated, and reported more sleep problems. The PSG data were collected during a single night only, and several studies^40,41^ have reported significant night-to-night variations in PSG-based OSA assessments, increasing data variance. However, multinight PSG measurements are scarce in common clinical and research settings and were also unavailable for the SHIP-Trend-0 study because of limited resources.^30^ In addition, no information on prior OSA diagnoses and previous or ongoing OSA treatments were available, possibly biasing the associations between OSA and WMHs detected in our study. Nevertheless, the prevalence of undiagnosed and thus untreated OSA is rather high in the general population,^42^ and we therefore expect only a few SHIP-Trend-0 study participants to be effectively undergoing OSA treatments.

## Conclusions

These analyses found significant associations between OSA, diagnosed by PSG, and brain WMHs in a large-scale, general population study. Future studies might investigate the effect of OSA on WMH burden in specific OSA populations and the effect of OSA treatments on WMH burden in longitudinal clinical trials.

## Notes

Supplement. **eMaterials and Methods.** Detailed Information About Data Assessment in SHIP-Trend-0, Magnetic Resonance Imaging Acquisition Parameters, and Categorization of Obstructive Sleep Apnea Parameters **eFigure 1.** Flowchart of Study Participant Exclusion Criteria in this Study **eFigure 2.** The Proportional Reduction in Sum of Squares After the Addition of Obstructive Sleep Apnea Parameters or Additional Metabolic, Vascular, and Lifestyle Risk Factors for White Matter Hyperintensities Volumes and Counts of the Null Model **eFigure 3.** Estimated Effect Sizes of Categorized Obstructive Sleep Apnea Parameters on White Matter Hyperintensities in Extended Regression Models **eTable 1.** Characteristics of Complete Study Sample vs Excluded SHIP-Trend-0 Subcohort **eTable 2.** Comparison of Study Sample Characteristics Between Subjects Without and With Obstructive Sleep Apnea **eTable 3.** Results of the Linear Regression Analysis for White Matter Hyperintensity Counts With Respect to Obstructive Sleep Apnea Diagnostic Criteria **eTable 4.** Results of the Linear Regression Analysis for White Matter Hyperintensities Volume and Count Data With Respect to Categorized Obstructive Sleep Apnea Diagnostic Criteria **eTable 5.** Results of the Linear Regression Analysis for White Matter Hyperintensities Volume and Count Data With Respect to Obstructive Sleep Apnea (OSA) Diagnostic Criteria After Extending the Respective OSA Models by Additional Metabolic, Vascular, and Lifestyle Covariates **eTable 6.** Results of the Linear Regression Analysis for White Matter Hyperintensities Volume and Count Data With Respect to Categorized Obstructive Sleep Apnea (OSA) Diagnostic Criteria After Extending the Respective OSA Models by Additional Metabolic, Vascular, and Lifestyle Covariates **eTable 7.** Results of the Linear Regression Analysis for White Matter Hyperintensity Volumes and Counts With Respect to an Interaction Term Between Obstructive Sleep Apnea Diagnostic Criteria and Different Metabolic, Vascular, and Lifestyle Risk Factors, Which Were Tested Separately in Individual Regression Models **eTable 8.** Results of the Linear Regression Analysis for Periventricular Frontal and Periventricular Dorsal White Matter Hyperintensity Volumes With Respect to Obstructive Sleep Apnea (OSA) Diagnostic Criteria After Extending the Respective OSA Models by Additional Metabolic, Vascular, and Lifestyle Covariates
