# Supplementary material for: Association Between Obstructive Sleep Apnea and Brain White Matter Hyperintensities in a Population-Based Cohort in Germany
Source: JAMA Netw Open. 2021 Oct 5;4(10):e2128225. doi: 10.1001/jamanetworkopen.2021.28225 (PMC8493431; doi:10.1001/jamanetworkopen.2021.28225)
Supplement: Supplement. — eMaterials and Methods. Detailed Information About Data Assessment in SHIP-Trend-0, Magnetic Resonance Imaging Acquisition Parameters, and Categorization of Obstructive Sleep Apnea Parameters eFigure 1. Flowchart of Study Participant Exclusion Criteria in this Study eFigure 2. The Proportional Reduction in Sum of Squares After the Addition of Obstructive Sleep Apnea Parameters or Additional Metabolic, Vascular, and Lifestyle Risk Factors for White Matter Hyperintensities Volumes and Counts of the Null Model eFigure 3. Estimated Effect Sizes of Categorized Obstructive Sleep Apnea Parameters on White Matter Hyperintensities in Extended Regression Models eTable 1. Characteristics of Complete Study Sample vs Excluded SHIP-Trend-0 Subcohort eTable 2. Comparison of Study Sample Characteristics Between Subjects Without and With Obstructive Sleep Apnea eTable 3. Results of the Linear Regression Analysis for White Matter Hyperintensity Counts With Respect to Obstructive Sleep Apnea Diagnostic Criteria eTable 4. Results of the Linear Regression Analysis for White Matter Hyperintensities Volume and Count Data With Respect to Categorized Obstructive Sleep Apnea Diagnostic Criteria eTable 5. Results of the Linear Regression Analysis for White Matter Hyperintensities Volume and Count Data With Respect to Obstructive Sleep Apnea (OSA) Diagnostic Criteria After Extending the Respective OSA Models by Additional Metabolic, Vascular, and Lifestyle Covariates eTable 6. Results of the Linear Regression Analysis for White Matter Hyperintensities Volume and Count Data With Respect to Categorized Obstructive Sleep Apnea (OSA) Diagnostic Criteria After Extending the Respective OSA Models by Additional Metabolic, Vascular, and Lifestyle Covariates eTable 7. Results of the Linear Regression Analysis for White Matter Hyperintensity Volumes and Counts With Respect to an Interaction Term Between Obstructive Sleep Apnea Diagnostic Criteria and Different Metabolic, Vascular, and Lifestyle Risk Fac [file jamanetwopen-e2128225-s001.pdf]

Supplementary Online Content

Zacharias HU, Weihs A, Habes M, et al. Association between obstructive sleep apnea and brain white matter hyperintensities in a population-based cohort in Germany. *JAMA Netw Open*. 2021;4(10):e2128225. doi:10.1001/jamanetworkopen.2021.28225

eMaterials and Methods. Detailed Information About Data Assessment in SHIP-Trend-0, Magnetic Resonance Imaging Acquisition Parameters, and Categorization of Obstructive Sleep Apnea Parameters
eFigure 1. Flowchart of Study Participant Exclusion Criteria in this Study
eFigure 2. The Proportional Reduction in Sum of Squares After the Addition of Obstructive Sleep Apnea Parameters or Additional Metabolic, Vascular, and Lifestyle Risk Factors for White Matter Hyperintensities Volumes and Counts of the Null Model
eFigure 3. Estimated Effect Sizes of Categorized Obstructive Sleep Apnea Parameters on White Matter Hyperintensities in Extended Regression Models
eTable 1. Characteristics of Complete Study Sample vs Excluded SHIP-Trend-0 Subcohort
eTable 2. Comparison of Study Sample Characteristics Between Subjects Without and With Obstructive Sleep Apnea
eTable 3. Results of the Linear Regression Analysis for White Matter Hyperintensity Counts With Respect to Obstructive Sleep Apnea Diagnostic Criteria
eTable 4. Results of the Linear Regression Analysis for White Matter Hyperintensities Volume and Count Data With Respect to Categorized Obstructive Sleep Apnea Diagnostic Criteria
eTable 5. Results of the Linear Regression Analysis for White Matter Hyperintensities Volume and Count Data With Respect to Obstructive Sleep Apnea (OSA) Diagnostic Criteria After Extending the Respective OSA Models by Additional Metabolic, Vascular, and Lifestyle Covariates
eTable 6. Results of the Linear Regression Analysis for White Matter Hyperintensities Volume and Count Data With Respect to Categorized Obstructive Sleep Apnea (OSA) Diagnostic Criteria After Extending the Respective OSA Models by Additional Metabolic, Vascular, and Lifestyle Covariates
eTable 7. Results of the Linear Regression Analysis for White Matter Hyperintensity Volumes and Counts With Respect to an Interaction Term Between Obstructive Sleep Apnea Diagnostic Criteria and Different Metabolic, Vascular, and Lifestyle Risk Factors, Which Were Tested Separately in Individual Regression Models
eTable 8. Results of the Linear Regression Analysis for Periventricular Frontal and Periventricular Dorsal White Matter Hyperintensity Volumes With Respect to Obstructive Sleep Apnea (OSA) Diagnostic Criteria After Extending the Respective OSA Models by Additional Metabolic, Vascular, and Lifestyle Covariates

This supplementary material has been provided by the authors to give readers additional information about their work.

© 2021 Zacharias HU et al. *JAMA Network Open.*

Supplementary Materials and Methods

Data assessment in SHIP-Trend-0

Glycated hemoglobin (HbA1c) values were determined by high-performance liquid chromatography (Bio-Rad Diamat). Serum high-density lipoprotein (HDL), low-density lipoprotein (LDL), triglyceride, high-sensitive C-reactive protein (hsCRP) as well as total cholesterol values were measured photometrically (Hitachi 704, Roche and Dimension VISTA, Siemens Healthineers, Erlangen, Germany). White blood cell (WBC) counts were determined in EDTA whole blood (XT 2000, XE 5000 or SE 9000, Sysmex, Deutschland GmbH, Norderstedt, Germany, or Adivia 2120i, Siemens Healthineers, Erlangen, Germany), and the fibrinogen concentrations were determined in citrate plasma according to Clauss (BCS or BCS XP, Siemens Healthineers, Erlangen, Germany). Smoking was divided into three categories: current smoking, former smoking, and never smoked. The education level was defined according to the number of school attendance years, i.e., < 10 years, 10 years, and > 10 years. Study participants were considered as physically active, if they carried out at least one hour of sports per week in winter and/or in summer. Alcohol consumption was based on self-reported intake of alcohol within the last month[1]. Diabetes was defined based on known diabetes diagnosed by a physician, the intake of antidiabetic drugs (HTC-code A10), an HbA1c value ≥ 6.5%, and/or a spontaneous blood sugar ≥ 11.1 mmol/l. Depression was based on the Composite International Diagnostic - Screener questionnaire[2,3].

After having completed the personal interview, study participants were medically examined, body height, weight, as well as hip and waist circumferences were measured, and the body-mass index (BMI) was calculated. After a 5 min resting phase, blood pressure was measured three times on the right arm of seated subjects with a digital blood pressure monitor (HEM-705 CP, Omron), and each reading being followed by a further resting period of 3 min. Cuffs were applied according to the circumference of the study participant's arm, and the mean of the second and third measurements was used for analyses. The intake of medications within the last 7 days was based on the packages

© 2021 Zacharias HU et al. *JAMA Network Open.*

brought to the interview by the study participants. We focused on antidiabetic, antihypertensive, as well as lipid lowering drugs, reflecting cardiovascular disease risk factor indicators in the general population. Hypertension was diagnosed if the systolic blood pressure ≥ 140 mmHg, if the diastolic blood pressure ≥ 90 mmHg, and/or if antihypertensive drugs had been prescribed by a physician within the last year. Mental health was assessed employing the SF-12 mental health summary scale[4–7].

Magnetic resonance imaging acquisition parameters

In brief, all images were acquired on a 1.5 T MRI scanner (Magnetom Avanto, Siemens Medical Systems, Erlangen, Germany) with an axial MPRAGE sequence and the following parameters: 1 x 1 mm in-plane spatial resolution, slice thickness = 1.0 mm (flip angle 15°), echo time = 3.4 ms and repetition time = 1,900 ms as well as axial T_2-FLAIR sequence with the following parameters: 0.9 x 0.9 mm in-plane spatial resolution, slice thickness = 3.0 mm (flip angle 15°), echo time = 325 ms and repetition time = 5,000 ms.

Categorization of obstructive sleep apnea parameters

OSA severity was categorized according to AHI values as follows: no OSA: AHI < 5/h of TST; mild OSA: AHI (5 – 15)/h of TST; moderate OSA: AHI (15 – 30)/h of TST; severe OSA: AHI ≥ 30/h of TST[8]. The oxygen desaturation index (ODI) was defined as the number of ≥ 4% oxygen desaturations per hour of TST determined by pulse oximetry. OSA severity according to ODI values was categorized as follows: no OSA: ODI < 5/h of TST; mild OSA: ODI (5 – 15)/h of TST; moderate OSA: ODI (15 – 30)/h of TST; severe OSA: ODI ≥ 30/h of TST[9].

References

1. Baumeister SE, Alte D, Meyer C, John U. Riskanter alkoholkonsum und alkoholbezogene störungen in Vorpommern: Die studie “leben und gesundheit in Vorpommern” (SHIP) und der

© 2021 Zacharias HU et al. *JAMA Network Open.*

Bundesgesundheitssurvey 1998 im vergleich. *Gesundheitswesen*. 2005. doi:10.1055/s-2004-813829

2. Wittchen MH, Gander F, Pfister H, et al. Screening for mental disorders: performance of the Composite International Diagnostic Screener (CID-S). *Int J Methods Psychiatr Res*. 1999;8(2):59-70.

3. Grabe HJ, Baumeister SE, John U, Freyberger HJ, Völzke H. Association of mental distress with health care utilization and costs: A 5-year observation in a general population. *Soc Psychiatry Psychiatr Epidemiol*. 2009;44(10):835-844. doi:10.1007/s00127-009-0005-9

4. Ware JE, Kosinski M, Keller SD. A 12-Item Short-Form Health Survey: Construction of Scales and Preliminary Tests of Reliability and Validity. *Med Care*. 1996. doi:10.1097/00005650-199603000-00003

5. Bullinger M, Kirchberger I, Ware J. Der deutsche SF-36 Health Survey Übersetzung und psychometrische Testung eines krankheitsübergreifenden Instruments zur Erfassung der gesundheitsbezogenen Lebensqualität. *Z Gesundh Wiss*. 1995. doi:10.1007/BF02959944

6. Bullinger M. German translation and psychometric testing of the SF-36 Health Survey: Preliminary results from the IQOLA project. *Soc Sci Med*. 1995. doi:10.1016/0277-9536(95)00115-N

7. Ware JE, Sherbourne CD. The MOS 36-item short-form health survey (SF-36). I. Conceptual framework and item selection. *Med Care*. 1992.

8. Stubbe B, Penzel T, Fietze I, et al. Polysomnography in a large population based study—the Study of Health in Pomerania protocol. 2016;2(1):1-5.

9. Senaratna C V., Lowe A, Perret JL, et al. Comparison of apnoea–hypopnoea index and oxygen desaturation index when identifying obstructive sleep apnoea using type-4 sleep studies. *J Sleep Res*. 2018;(October):1-8. doi:10.1111/jsr.12804

© 2021 Zacharias HU et al. *JAMA Network Open.*

Supplementary Figures

Supplementary Figure S1. Flowchart of study participant exclusion criteria in this study. Polysomnography (PSG) data was available for 1,109 study participants of SHIP-Trend-0. White matter hyperintensity (WMH) and intracranial volume (ICV) data were available from 1,959 and 1,907 SHIP-Trend-0 study participants, respectively. In total, WMH, ICV, and PSG data were available for 607 study participants. Further SHIP-Trend-0 study participant exclusions had to be carried out due to low quality WMH data (*n* = 29), missing phenodata (diabetes status, glycated hemoglobin, depression status, physical activity parameters, hypertension status, blood pressure, smoking status, education status, SF-12 questionnaire evaluation, high-resolution C-reactive protein, alcohol consumption; *n* = 46), and after assessment of the residual normality (*n* = 3). For the analyses of regional WMH volume data, 392 SHIP-Trend-0 study participants could be included, since remaining study participants (*n* = 137) had to be excluded due to missing regional WMH volume data.

© 2021 Zacharias HU et al. *JAMA Network Open.*

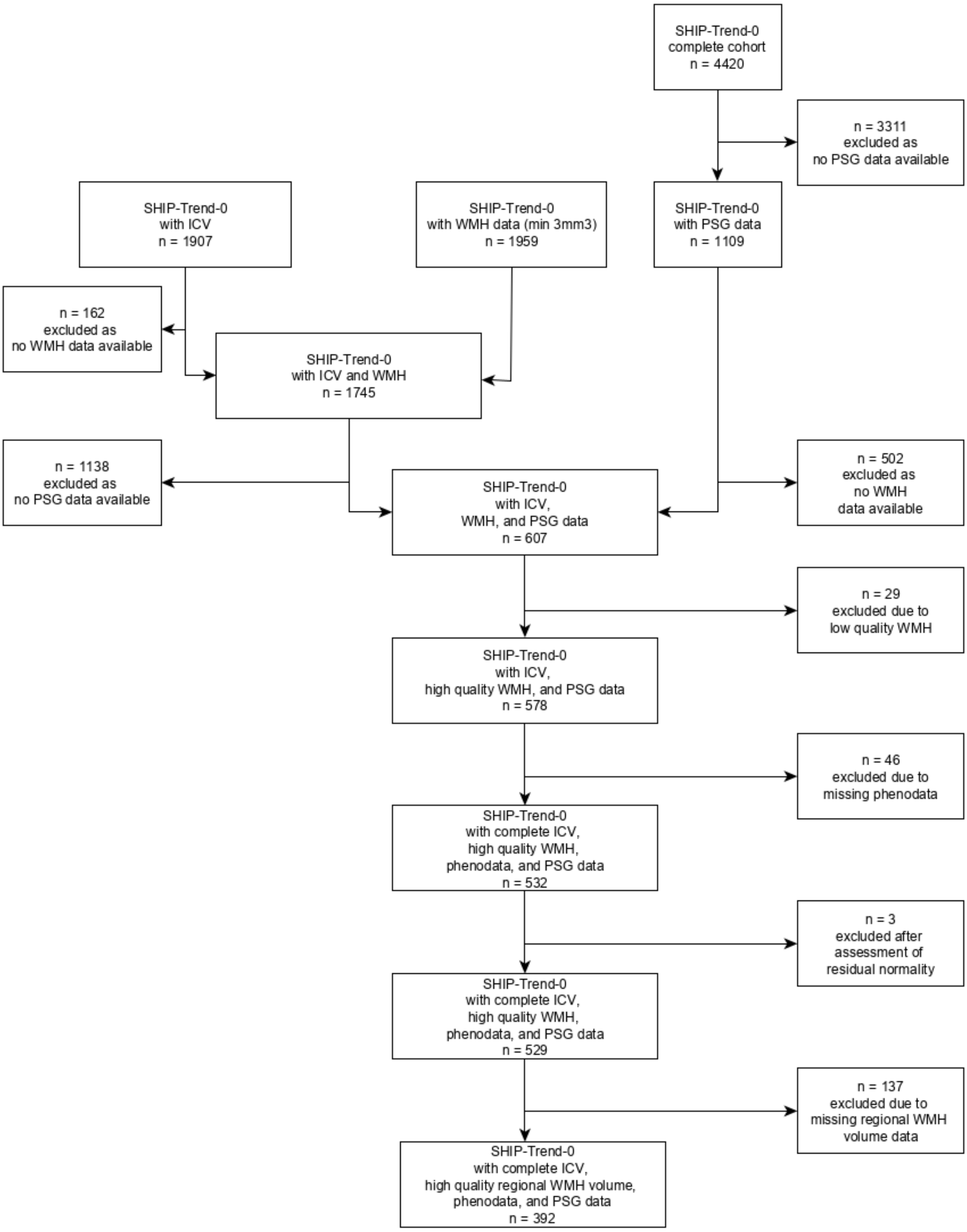

© 2021 Zacharias HU et al. *JAMA Network Open.*

Supplementary Figure S2. The proportional reduction in sum of squares after the addition of OSA parameters or additional metabolic, vascular, and lifestyle risk factors for (a) white matter hyperintensities (WMH) volumes and (b) counts in a one-by-one fashion, as indicated on the *y*-axis, to the null model comprising sex, age modelled by a restricted cubic spline, intracranial volume, and body height. Corresponding *F* statistics (*p*-values for comparisons between the extended model against the null model) are given on the right side of each plot. Abbreviations: AHI, apnea-hypopnea index; HDL, high-density lipoprotein; LDL, low-density lipoprotein; ODI, oxygen desaturation index; OSA_AHI, categorized obstructive sleep apnea defined according to AHI; OSA_ODI, categorized obstructive sleep apnea defined according to ODI.

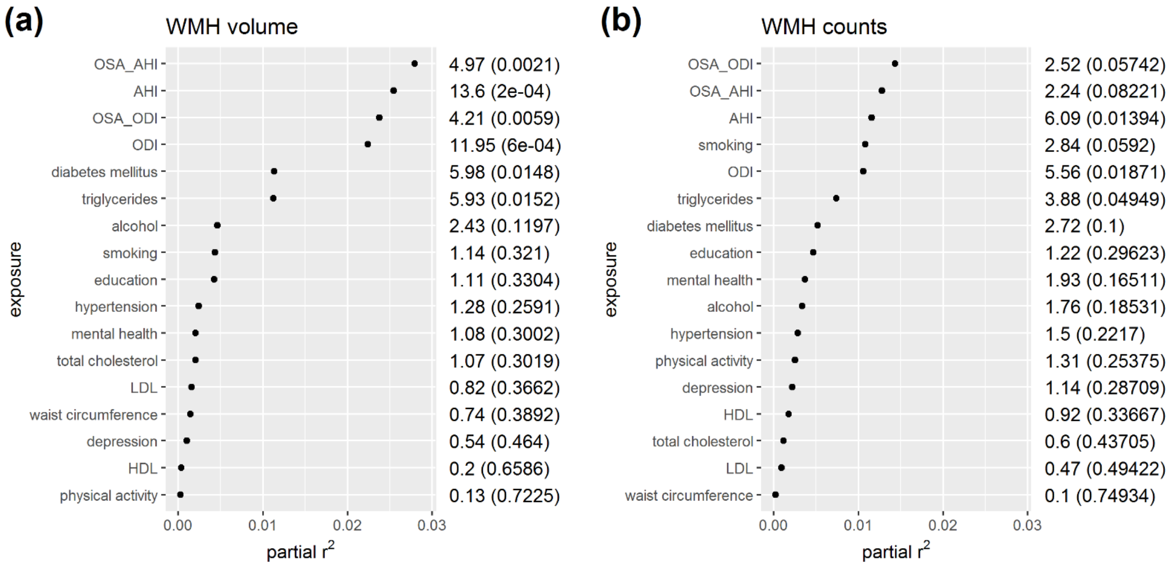

© 2021 Zacharias HU et al. *JAMA Network Open.*

Supplementary Figure S3. Estimated effect sizes of categorized obstructive sleep apnea (OSA) parameters on white matter hyperintensities (WMH) in extended regression models. The *x*-axis gives the estimated effect sizes (regression coefficients *β*) and 95% confidence intervals of obstructive sleep apnea defined by either apnea-hypopnea index (OSA_{AHI}), or oxygen-desaturation index (OSA_{ODI}), on (a) white matter hyperintensity (WMH) volumes and (b) counts after extension of the OSA regression models by the additional confounder variables given on the *y*-axis. The *β*-coefficients are either represented as circles in the case of significant associations (*p*-value < .05) between the respective OSA parameter and WMH, or triangles in the case of non-significant associations (*p*-value ≥ .05). Dashed vertical lines mark the respective OSA *β*-coefficients in the OSA models only adjusted for sex, age modelled by a restricted cubic spline, intracranial volume, and body height. The full model includes the respective OSA parameter, the complete set of metabolic, vascular, and lifestyle covariates, as well as the null model covariates. No OSA was considered as the reference category. Mild, moderate, and severe OSA_{AHI} *β*-coefficients are plotted in red, green, and blue, respectively. Mild, moderate, and severe OSA_{ODI} *β*-coefficients are plotted in olive green, turquoise, and violet, respectively. Abbreviations: AHI, apnea-hypopnea index, HDL, high-density lipoprotein; LDL, low-density lipoprotein; ODI, oxygen desaturation index; OSA, obstructive sleep apnea; WMH, white matter hyperintensities.

© 2021 Zacharias HU et al. *JAMA Network Open.*

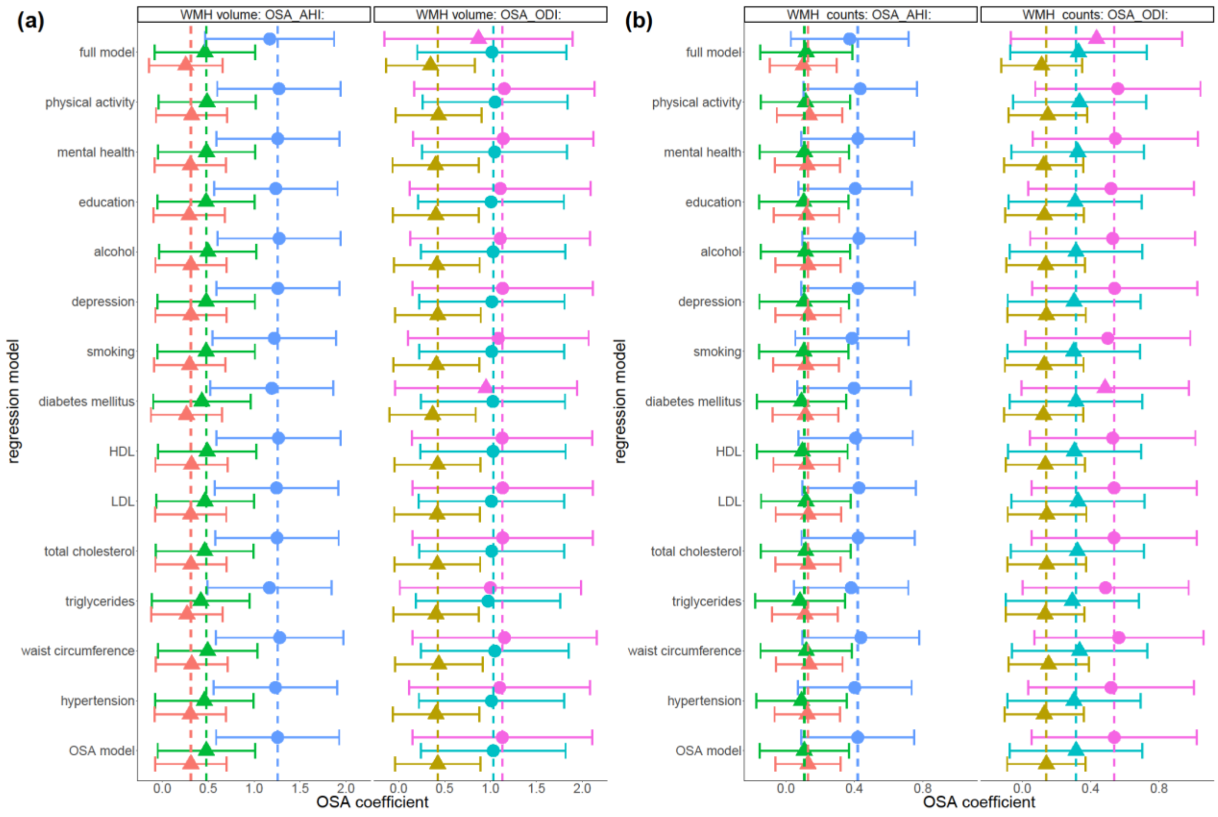

© 2021 Zacharias HU et al. *JAMA Network Open.*

Supplementary Tables

Supplementary Table S1. Characteristics of complete study sample versus excluded SHIP-Trend-0 subcohort. Abbreviations: AHI, apnea hypopnea index; BMI, body-mass index; d, days; IQR, interquartile range; NA, not assigned value; NREM, non- Rapid Eye Movement sleep; OS, oxygen saturation; REM, Rapid Eye Movement sleep; SD, standard deviation; T1DM, type-1 diabetes mellitus; T2DM, type-2 diabetes mellitus; TST, total sleep time. [a]*P*-values for categorical variables were calculated using Pearson's Chi-squared test, *p*-values for continuous variables were calculated using a two-sided *t*-test assuming unequal variance. *Significance level at $p < 0.05$.

	**SHIP-Trend-0 included**	**Number of NAs in SHIP-Trend-0 included**	**SHIP-Trend-0 excluded**	**Number of NAs in SHIP-Trend-0 excluded**	***p*-value[a]**
	**(*n* = 529)**		**(*n* = 3,891)**		
**Demographic and clinical chemistry parameters:**					
	**mean (SD)**	***n***	**mean (SD)**	***n***	
**Age, years**	52.15 (13.58)	0	51.93 (15.70)	0	.74
**Systolic blood pressure, mmHg**	126.83 (16.98)	0	128.14 (18.90)	19	.10
**Diastolic blood pressure, mmHg**	77.36 (9.44)	0	77.27 (10.36)	20	.84
**Glycated hemoglobin (HbA1c), %**	5.33 (0.73)	0	5.38 (0.85)	9	.22
**Total cholesterol, mmol/l**	5.56 (1.09)	0	5.43 (1.14)	7	.01*
**Total triglycerides, mmol/l**	1.58 (1.12)	0	1.69 (1.23)	7	.05*
**High-density lipoprotein, mmol/l**	1.44 (0.35)	0	1.43 (0.39)	7	.56
**Low-density lipoprotein, mmol/l**	3.48 (0.92)	0	3.33 (0.97)	7	< .001*
**Waist circumference, cm**	90.16 (12.78)	0	91.40 (14.78)	14	.04*
**Hip circumference, cm**	102.28 (9.17)	0	102.67 (10.95)	9	.37
**Body height, cm**	169.87 (9.08)	0	169.82 (9.41)	7	.91
**Body weight, kg**	80.90 (14.62)	0	81.28 (17.18)	5	.58
**BMI, kg/m²**	27.99 (4.34)	0	28.14 (5.34)	7	.48
**C-reactive protein, mg/l**	2.61 (5.12)	0	2.62 (4.92)	199	.98
**Fibrinogen, g/l**	3.08 (0.74)	5	3.07 (0.74)	43	.79
**White blood cell counts, Gpt/l**	5.81 (2.71)	1	6.22 (2.53)	14	.001*
**Alcohol within last 30d, g/d**	8.65 (11.88)	0	73.46 (2,806.67)	20	.15
**Subjective mental health summary scale**	52.98 (8.50)	0	52.37 (8.63)	110	.12
	***n* (%)**	***n***	***n* (%)**	***n***	
**Sex, women**	282 (53)	0	1,993 (51)	0	.39
**Diabetes (T1DM or T2DM)**	53 (10)	0	497 (13)	20	.08
**Cigarette smoking**		0		22	< .001*
Never-smoker	233 (44)		1,372 (35)		
Ex-smoker	202 (38)		1,408 (36)		
Current smoker	94 (18)		1,089 (28)		
**Physical activity**	392 (74)	0	2,646 (68)	22	.009*
**Education**		0		13	< .001*
< 10 years	71 (13)		963 (25)		
10 years	278 (53)		1,987 (51)		
> 10 years	180 (34)		928 (24)		
**Lifetime Depression Medication**	163 (31)	0	1,061 (28)	92	.18

© 2021 Zacharias HU et al. *JAMA Network Open.*

Anti-diabetic drugs	25 (5)	0	322 (8)	0	.006*
Antihypertensive drugs	184 (35)	0	1,524 (39)	0	0.06
Lipid lowering drugs	48 (9)	0	573 (15)	0	< .001*
**Hypertension**	240 (45)	0	1,875 (48)	21	.12
**Sleep questionnaire parameters:**					
	***n* (%)**	***n***	***n* (%)**	***n***	
**Sleep time normal work day, mean (SD), h**	6.92 (1.26)	0	8.85 (42.16)	27	.005*
**No nap in last 7d**	307 (58)	0	2,147 (56)	27	.31
**How often > 30min for falling asleep per week?**		0		27	.38
4 - 7 times	104 (20)		681 (18)		
1 - 3 times	116 (22)		802 (21)		
< 1 time per week	84 (16)		560 (14)		
no problems falling asleep	224 (42)		1,815 (47)		
don't know	1 (0)		6 (0)		
**How often awake during night > 30min per week?**		0		27	.01*
4 - 7 times	64 (12)		401 (10)		
1 - 3 times	81 (15)		549 (14)		
< 1 time per week	83 (16)		424 (11)		
no problems staying asleep	134 (25)		1,144 (30)		
often wakes up during night, but gets back to sleep	166 (31)		1,343 (35)		
don't know	1 (0)		2 (0)		
answer refused	0 (0)		1 (0)		
**snoring**		0		27	< .001*
regularly	141 (27)		732 (19)		
occasionally	245 (46)		1,811 (47)		
never	106 (20)		1,082 (28)		
don't know	37 (7)		239 (6)		
**Magnetic resonance imaging parameters:**					
	**mean (SD)**	***n***	**mean (SD)**	***n***	
**Intracranial volume, mm³**	1,581,582.54 (155,427.52)	0	1,581,575.48 (154,957.24)	2513	> .99
**WMH volume, median (IQR), mm³**	208 (83 - 590)	0	200.5 (76 - 519)	2461	.07
**WMH counts**	13.55 (10.61)	0	13.96 (12.81)	2461	.48

© 2021 Zacharias HU et al. *JAMA Network Open.*

Supplementary Table S2. Comparison of study sample characteristics between subjects without and with obstructive sleep apnea (OSA). (a) OSA defined according to apnea-hypopnea index (AHI) criteria, and (b) OSA defined according to oxygen desaturation index (ODI) criteria. Abbreviations: AHI: apnea-hypopnea index, ODI: oxygen desaturation index, OSA: obstructive sleep apnea, TST: total sleep time. [a]*P*-values for categorical variables were calculated using Pearson's Chi-squared test, *p*-values for continuous variables were calculated using a two-sided *t*-test assuming unequal variance. *Significance level at $p < .05$.

**(a) OSA defined according to AHI criteria.**			
	**No OSA group (AHI < 5h/TST; *n* = 320)**	**OSA group (AHI ≥ 5 h/TST; *n* = 209)**	***P*-value[a]**
**Demographic and clinical chemistry parameters:**			
	**mean (SD)**	**mean (SD)**	
**Age, years**	47.91 (13.80)	58.64 (10.31)	< .001*
**Systolic blood pressure, mmHg**	122.69 (15.68)	133.17 (16.96)	< .001*
**Diastolic blood pressure, mmHg**	75.64 (8.52)	79.98 (10.18)	< .001*
**Glycated hemoglobin (HbA1c), %**	5.17 (0.63)	5.59 (0.79)	< .001*
**Total cholesterol, mmol/l**	5.49 (1.11)	5.67 (1.05)	.06
**Total triglycerides, mmol/l**	1.38 (0.89)	1.90 (1.34)	< .001*
**High-density lipoprotein, mmol/l**	1.52 (0.37)	1.33 (0.29)	< .001*
**Low-density lipoprotein, mmol/l**	3.38 (0.94)	3.63 (0.86)	.002*
**Waist circumference, cm**	85.85 (11.80)	96.76 (11.36)	< .001*
**Hip circumference, cm**	100.48 (8.94)	105.04 (8.83)	< .001*
**Body height, cm**	169.78 (9.07)	169.99 (9.13)	.80
**Body weight, kg**	77.21 (13.76)	86.54 (14.13)	< .001*
**BMI, kg/m²**	26.74 (4.03)	29.91 (4.10)	< .001*
**C-reactive protein, mg/l**<br>**Fibrinogen, g/l**<br>**White blood cell counts, Gpt/l**	2.20 (3.02)<br>2.97 (0.65)<br>5.65 (1.54)	3.24 (7.20)<br>3.25 (0.83)<br>6.07 (3.87)	.05*<br>< .001*<br>.14
**Alcohol within last 30d, g/d**	7.90 (11.49)	9.79 (12.39)	.08
**Subjective mental health summary scale**	52.52 (8.37)	53.67 (8.66)	.13
	***n* (%)**	***n* (%)**	
**Sex, women**	199 (62)	83 (40)	< .001*
**Diabetes (T1DM or T2DM)**	16 (5)	37 (18)	< .001*
**Cigarette smoking**			.002*
Never-smoker	151 (47)	82 (39)	
Ex-smoker	103 (32)	99 (47)	
Current smoker	66 (21)	28 (13)	
**Physical activity**	244 (76)	148 (71)	.20
**Education**			.004*
< 10 years	31 (10)	40 (19)	
10 years	170 (53)	108 (52)	
> 10 years	119 (37)	61 (29)	
**Lifetime Depression**	105 (33)	58 (28)	.26
**Medication**			
Anti-diabetic drugs	7 (2)	18 (9)	.001*
Antihypertensive drugs	79 (25)	105 (50)	< .001*
Lipid lowering drugs	18 (6)	30 (14)	.001*
**Hypertension**	108 (34)	132 (63)	< .001*

© 2021 Zacharias HU et al. *JAMA Network Open.*

Sleep questionnaire parameters:			
	***n* (%)**	***n* (%)**	
**Sleep time normal work day, mean (SD), h**	6.88 (1.27)	7.00 (1.25)	.28
**No nap in last 7d**	213 (67)	94 (45)	< .001*
**How often > 30min for falling asleep per week?**			.28
4 - 7 times	62 (19)	42 (20)	
1 - 3 times	65 (20)	51 (24)	
< 1 time per week	58 (18)	26 (12)	
no problems falling asleep	135 (42)	89 (43)	
don't know	0 (0)	1 (0)	
**How often awake during night > 30min per week?**			.36
4 - 7 times	43 (13)	21 (10)	
1 - 3 times	43 (13)	38 (18)	
< 1 time per week	49 (15)	34 (16)	
no problems staying asleep	86 (27)	48 (23)	
often wakes up during night, but gets back to sleep	99 (31)	67 (32)	
don't know	0 (0)	1 (0)	
answer refused	0 (0)	0 (0)	
**Snoring**			< .001*
regularly	57 (18)	84 (40)	
occasionally	143 (45)	102 (49)	
never	92 (29)	14 (7)	
don't know	28 (9)	9 (4)	
**Magnetic resonance imaging parameters:**			
	**mean (SD)**	**mean (SD)**	
**Intracranial volume, mm³**	$1.57e^6$ ($0.15e^6$)	$1.60e^6$ ($0.16e^6$)	.06

(b) OSA defined according to ODI criteria.			
	**No OSA group (ODI < 5h/TST; *n* = 427)**	**OSA group (ODI ≥ 5h/TST; *n* = 102)**	***P*-value[a]**
**Demographic and clinical chemistry parameters:**			
	**mean (SD)**	**mean (SD)**	
**Age, years**	50.15 (13.52)	60.49 (10.32)	< .001*
**Systolic blood pressure, mmHg**	124.64 (16.14)	135.98 (17.39)	< .001*
**Diastolic blood pressure, mmHg**	76.29 (8.66)	81.80 (11.18)	< .001*
**Glycated hemoglobin (HbA1c), %**	5.24 (0.67)	5.71 (0.84)	< .001*
**Total cholesterol, mmol/l**	5.53 (1.11)	5.66 (0.99)	.24
**Total triglycerides, mmol/l**	1.50 (0.99)	1.93 (1.50)	.007*
**High-density lipoprotein, mmol/l**	1.47 (0.36)	1.31 (0.30)	< .001*
**Low-density lipoprotein, mmol/l**	3.44 (0.94)	3.65 (0.82)	.02*
**Waist circumference, cm**	87.70 (11.82)	100.46 (11.51)	< .001*
**Hip circumference, cm**	101.22 (9.01)	106.73 (8.52)	< .001*
**Body height, cm**	169.77 (9.15)	170.26 (8.85)	.62
**Body weight, kg**	78.68 (13.71)	90.21 (14.72)	< .001*
**BMI, kg/m²**	27.26 (4.09)	31.04 (4.03)	< .001*
**C-reactive protein, mg/l**<br>**Fibrinogen, g/l**<br>**White blood cell counts, Gpt/l**	2.39 (4.55)<br>3.04 (0.72)<br>5.67 (1.56)	3.56 (6.94)<br>3.26 (0.79)<br>6.42 (5.28)	.11<br>.01*<br>.16
**Alcohol within last 30d, g/d**	8.06 (11.21)	11.11 (14.14)	.04*
**Subjective mental health summary scale**	52.86 (8.48)	53.46 (8.60)	.53
	***n* (%)**	***n* (%)**	
**Sex, women**	248 (58)	34 (33)	< .001*
**Diabetes (T1DM or T2DM)**	30 (7)	23 (23)	< .001*

© 2021 Zacharias HU et al. *JAMA Network Open.*

**Cigarette smoking**			.006*
Never-smoker	197 (46)	36 (35)	
Ex-smoker	149 (35)	53 (52)	
Current smoker	81 (19)	13 (13)	
**Physical activity**	323 (76)	69 (68)	.13
**Education**			< .001*
< 10 years	46 (11)	25 (25)	
10 years	228 (53)	50 (49)	
> 10 years	153 (36)	27 (26)	
**Lifetime Depression**	139 (33)	24 (24)	.10
**Medication**			
Anti-diabetic drugs	15 (4)	10 (10)	.02*
Antihypertensive drugs	125 (29)	59 (58)	< .001*
Lipid lowering drugs	31 (7)	17 (17)	.005*
**Hypertension**	165 (39)	75 (74)	< .001*
**Sleep questionnaire parameters:**			
	***n* (%)**	***n* (%)**	
**Sleep time normal work day, mean (SD), h**	6.88 (1.26)	7.12 (1.26)	.08
**No nap in last 7d**	267 (63)	40 (39)	< .001*
**How often > 30min for falling asleep per week?**			
4 - 7 times	88 (21)	16 (16)	
1 - 3 times	91 (21)	25 (25)	
< 1 time per week	70 (16)	14 (14)	
no problems falling asleep	178 (42)	46 (45)	
don't know	0 (0)	1 (1)	
**How often awake during night > 30min per week?**			.19
4 - 7 times	55 (13)	9 (9)	
1 - 3 times	64 (15)	17 (17)	
< 1 time per week	66 (15)	17 (17)	
no problems staying asleep	103 (24)	31 (30)	
often wakes up during night, but gets back to sleep	139 (33)	27 (26)	
don't know	0 (0)	1 (1)	
answer refused	0 (0)	0 (0)	
**snoring**			< .001*
regularly	92 (22)	49 (48)	
occasionally	205 (48)	40 (39)	
never	99 (23)	7 (7)	
don't know	31 (7)	6 (6)	
**Magnetic resonance imaging parameters:**			
	**mean (SD)**	**mean (SD)**	
**Intracranial volume, mm^3**	$1.58e^6$ ($0.15e^6$)	$1,61e^6$ ($0.17e^6$)	.09

© 2021 Zacharias HU et al. *JAMA Network Open.*

Supplementary Table S3. Results of the linear regression analysis for white matter hyperintensity (WMH) counts with respect to obstructive sleep apnea diagnostic criteria. The null model regressed the independent variables WMH counts on the explanatory variables sex, age modelled by a restricted cubic spline, intracranial volume, and body height. The OSA model extended the explanatory variables of the null model by either (a) apnea-hypopnea (AHI) or (b) oxygen desaturation index (ODI), respectively. Abbreviations: AHI, apnea-hypopnea index; df, degrees of freedom; ICV, intracranial volume; ODI, oxygen desaturation index; rcs, restricted cubic spline; se, standard error. **P*-value < .05; [a]WMH count data has been log_2-transformed after the addition of a pseudocount of 1; [b]*p*-values were calculated using a Wald test.

(a) AHI regression models

	WMH count[a]					
	Null model			AHI model		
	β **(se)**	***p*-value**		***β*** **(se)**	***p*-value**	
**Constant**	-1.626 (1.194)	.18		-1.649 (1.189)	.17	
**AHI**				0.008 (0.003)	.01*	
**Female sex**	-0.238 (0.111)	.03*		-0.183 (0.113)	.11	
**rcs age**	0.047 (0.010)	< .001*		0.045 (0.010)	< .001*	
**rcs age'**	0.013 (0.022)	.56	< .001*[b]	0.012 (0.022)	.58	< .001*[b]
**rcs age''**	-0.105 (0.110)	.34		-0.103 (0.110)	.35	
**ICV**	$1e^{-6}$ ($3e^{-7}$)	< .001*		$1e^{-6}$ ($3e^{-7}$)	< .001*	
**Body height**	0.003 (0.006)	.62		0.003 (0.006)	.62	
**Observations**	529					
R²	0.390			0.397		
**Adjusted *R²***	0.383			0.389		
**Residual Std. Error**	0.854 (df = 522)			0.850 (df = 521)		

(b) ODI regression models

	WMH count[a]					
	Null model			ODI model		
	β **(se)**	***p*-value**		***β*** **(se)**	***p*-value**	
**Constant**	-1.626 (1.194)	.18		-1.772 (1.191)	.14	
**ODI**				0.011 (0.005)	.02*	
**Female sex**	-0.238 (0.111)	.03*		-0.187 (0.113)	.10	
**rcs age**	0.047 (0.010)	< .001*		0.046 (0.010)	$2e^{-5}$*	
**rcs age'**	0.013 (0.022)	.56	< .001*[a]	0.012 (0.022)	0.574	< .001*[a]
**rcs age''**	-0.105 (0.110)	.34		-0.105 (0.110)	0.340	
**ICV**	$1e^{-6}$ ($3e^{-7}$)	< .001*		$1e^{-6}$ ($3e^{-7}$)	< .001*	
**Body height**	0.003 (0.006)	.62		0.004 (0.006)	.57	
**Observations**	529					
R²	0.390			0.396		
**Adjusted *R²***	0.383			0.388		
**Residual Std. Error**	0.854 (df = 522)			0.851 (df = 521)		

© 2021 Zacharias HU et al. *JAMA Network Open.*

Supplementary Table S4. Results of the linear regression analysis for white matter hyperintensities (WMH) volume and count data with respect to categorized obstructive sleep apnea (OSA) diagnostic criteria. OSA diagnosed according to the apnea-hypopnea index (OSA_{AHI}) was categorized as follows: no OSA: AHI < 5/h of total sleep time (TST); mild OSA: AHI (5 – 15)/h of TST; moderate OSA: AHI (15 – 30)/h of TST; severe OSA: AHI ≥ 30/h of TST. OSA diagnosed according to the oxygen desaturation index (OSA_{ODI}) was categorized as follows: no OSA: ODI < 5/h of TST; mild OSA: ODI (5 – 15/h of TST; moderate OSA: ODI (15 – 30)/h of TST; severe OSA: ODI ≥ 30/h of TST. No OSA was considered as the reference category. The null model regressed the independent variables WMH volumes or counts on the explanatory variables sex, age modelled by a restricted cubic spline, intracranial volume, and body height. The OSA model extended the explanatory variables of the null model by either categorized (a) OSA_{AHI} or (b) OSA_{ODI}, respectively. Abbreviations: AHI, apnea-hypopnea index; df, degrees of freedom ICV, intracranial volume; ODI, oxygen desaturation index; rcs, restricted cubic spline; se, standard error; df, degrees of freedom. *P-value < .05; [a]WMH volume and count data have been $\log_2$-transformed after the addition of a pseudocount of 1; [b]p-values were calculated using a Wald test.

(a) AHI regression models												
	WMH volume[a]						WMH count[a]					
	Null model			AHI model			Null model			AHI model		
	β (se)	*p*-value		*β* (se)	*p*-value		*β* (se)	*p*-value		*β* (se)	*p*-value	
**Constant**	-0.581 (2.438)	.81		-0.700 (2.413)	.77		-1.626 (1.194)	.18		-1.690 (1.191)	.16	
**AHI**												
mild OSA_{AHI}				0.312 (0.197)	.12					0.129 (0.097)	.18	
moderate OSA_{AHI}				0.480 (0.270)	.08					0.107 (0.133)	.42	
severe OSA_{AHI}				1.255 (0.341)	< .001*					0.419 (0.168)	.01*	
**Sex**	-0.232 (0.227)	.31		-0.041 (0.231)	.86		-0.238 (0.111)	.03*		-0.172 (0.114)	.13	
**rcs age**	0.089 (0.021)	< .001*		0.081 (0.021)	< .001*		0.047 (0.010)	< .001*		0.044 (0.011)	< .001*	
**rcs age'**	0.031 (0.045)	.50	< .001*[b]	0.030 (0.045)	.50	< .001*[b]	0.013 (0.022)	.56	< .001*[b]	0.014 (0.022)	.54	< .001*[b]
**rcs age''**	-0.112 (0.225)	.62		-0.112 (0.223)	.62		-0.105 (0.110)	.34		-0.109 (0.110)	.33	
**ICV**	$3e^{-6}$ ($1e^{-6}$)	< .001*		$3e^{-6}$ ($1e^{-6}$)	< .001*		$1e^{-6}$ ($3e^{-7}$)	< .001*		$1e^{-6}$ ($3e^{-7}$)	< .001*	
**Body height**	-0.011 (0.013)	.38		-0.010 (0.013)	0.43		0.003 (0.006)	.62		0.004 (0.006)	.55	

© 2021 Zacharias HU et al. *JAMA Network Open.*

**Observations**	529			
R²	0.425	0.441	0.390	0.398
**Adjusted *R²***	0.418	0.431	0.383	0.387
**Residual Std. Error**	1.744 (df = 522)	1.724 (df = 519)	0.854 (df = 522)	0.851 (df = 519)

**(b) ODI regression models**												
	**WMH volume[a]**						**WMH count[a]**					
	**Null model**			**ODI model**			**Null model**			**ODI model**		
	***β* (se)**	***p*-value**		***β* (se)**	***p*-value**		***β* (se)**	***p*-value**		***β* (se)**	***p*-value**	
**Constant**	-0.581 (2.438)	.81		-0.805 (2.417)	.74		-1.626 (1.194)	.18		-1.695 (1.190)	.16	
**ODI**												
mild OSA_{ODI}				0.426 (0.237)	.07					0.141 (0.117)	.23	
moderate OSA_{ODI}				1.030 (0.401)	.01*					0.315 (0.197)	.11	
severe OSA_{ODI}				1.130 (0.499)	.02*					0.538 (0.246)	.03*	
**Sex**	-0.232 (0.227)	.31		-0.091 (0.229)	.69		-0.238 (0.111)	.03*		-0.186 (0.113)	.10	
**rcs age**	0.089 (0.021)	< .001*	< .001*[b]	0.085 (0.021)	< .001*	< .001*[b]	0.047 (0.010)	< .001*	< .001*[b]	0.046 (0.010)	< .001*	< .001*[b]
**rcs age'**	0.031 (0.045)	.50		0.029 (0.045)	.52		0.013 (0.022)	.56		0.012 (0.022)	.58	
**rcs age''**	-0.112 (0.225)	.62		-0.108 (0.223)	.63		-0.105 (0.110)	.34		-0.106 (0.110)	.34	
**ICV**	$3e^{-6}$ ($1e^{-6}$)	< .001*		$4e^{-6}$ ($1e^{-6}$)	< .001*		$1e^{-6}$ ($3e^{-7}$)	< .001*		$1e^{-6}$ ($3e^{-7}$)	< .001*	
**Body height**	-0.011 (0.013)	.38		-0.010 (0.013)	.43		0.003 (0.006)	.62		0.003 (0.006)	.59	
**Observations**	529											
R²	0.425			0.438			0.390			0.398		
**Adjusted *R²***	0.418			0.429			0.383			0.388		
**Residual Std. Error**	1.744 (df = 522)			1.728 (df = 519)			0.854 (df = 522)			0.851 (df = 519)		

© 2021 Zacharias HU et al. *JAMA Network Open.*

Supplementary Table S5. Results of the linear regression analysis for white matter hyperintensities (WMH) volume and count data with respect to obstructive sleep apnea (OSA) diagnostic criteria (a) apnea-hypopnea index (AHI) and (b) oxygen-desaturation index (ODI) after extending the respective OSA models by additional metabolic, vascular, and lifestyle covariates. For each new covariates model, the estimated effect sizes (*β*-coefficients), standard errors (se), and *p*-values of both the respective OSA parameter and the considered additional covariate are given. All models were adjusted for sex, age modelled by a restricted cubic spline, intracranial volume, and body height and are based on the complete study sample of 529 SHIP-Trend-0 study participants. Abbreviations: AHI, apnea-hypopnea index; ICV, intracranial volume; HDL, high-density lipoprotein; LDL, low-density lipoprotein; ODI, oxygen desaturation index; OSA, obstructive sleep apnea; rcs, restricted cubic spline; se, standard error. **P*-value < .05; [a]WMH volume and count data have been log_2-transformed after the addition of a pseudocount of 1.

(a) AHI regression models										
	WMH volume[a]					WMH count[a]				
	AHI		covariate		adjusted R^2	AHI		covariate		adjusted R^2
	β (se)	*p*-value	*β* (se)	*p*-value		*β* (se)	*p*-value	*β* (se)	*p*-value	
**OSA model**	0.024 (0.006)	< .001*			0.432	0.008 (0.003)	.01*			0.389
**Hypertension**	0.023 (0.007)	< .001*	0.103 (0.170)	.55	0.431	0.007 (0.003)	.02*	0.073 (0.084)	.38	0.388
**Waist circumference**	0.024 (0.007)	< .001*	-0.001 (0.007)	.84	0.431	0.008 (0.003)	.01*	-0.001 (0.004)	.69	0.388
**Triglycerides**	0.022 (0.007)	.002*	0.128 (0.071)	.07	0.434	0.007 (0.003)	.03*	0.054 (0.035)	.12	0.390
**Total cholesterol**	0.024 (0.007)	< .001*	0.062 (0.073)	.40	0.431	0.008 (0.003)	.01*	-0.033 (0.036)	.36	0.388
**LDL**	0.024 (0.007)	< .001*	0.054 (0.085)	.53	0.431	0.008 (0.003)	.01*	-0.037 (0.042)	.38	0.388
**HDL**	0.024 (0.007)	< .001*	0.023 (0.244)	.93	0.431	0.008 (0.003)	.02*	-0.074 (0.120)	.54	0.388
**Diabetes mellitus**	0.023 (0.006)	< .001*	0.557 (0.256)	.03*	0.436	0.008 (0.003)	.02*	0.185 (0.127)	.15	0.390
**Smoking**	0.023 (0.007)	< .001*			0.431	0.007 (0.003)	.02*			0.392
Ex-smoking			0.170 (0.170)	.32				0.151 (0.084)	.07	
Current smoking			0.227 (0.220)	.30				0.199 (0.108)	.07	
**Depression**	0.024 (0.006)	< .001*	-0.129 (0.171)	.45	0.431	0.008 (0.003)	.01*	-0.091 (0.084)	.28	0.389
**Alcohol**	0.024 (0.006)	< .001*	0.012 (0.007)	.09	0.434	0.008 (0.003)	.01*	0.005 (0.003)	.16	0.390
**Education**	0.024 (0.007)	< .001*			0.431	0.008 (0.003)	.02*			0.389
10 years			-0.193 (0.261)	.46				-0.043 (0.129)	.74	

> 10 years			-0.326 (0.264)	.22				-0.142 (0.130)	.28	
**Mental health**	0.024 (0.006)	< .001*	-0.010 (0.009)	.27	0.432	0.008 (0.003)	.01*	-0.006 (0.005)	.15	0.390
**Physical activity**	0.024 (0.007)	< .001*	0.114 (0.174)	.51	0.431	0.008 (0.003)	.01*	0.116 (0.086)	.18	0.390
**Full model**	0.022 (0.007)	.002*			0.435	0.007 (0.003)	.04*			0.398
Hypertension			0.095 (0.180)	.60				0.071 (0.088)	.43	
Waist circumference			-0.010 (0.009)	.26				-0.006 (0.004)	.17	
Triglycerides			0.206 (0.118)	.08				0.115 (0.058)	.05*	
Total cholesterol			-0.287 (0.289)	.32				-0.214 (0.142)	.13	
LDL			0.354 (0.305)	.25				0.178 (0.150)	.24	
HDL			0.412 (0.427)	.34				0.177 (0.210)	.40	
Diabetes mellitus			0.529 (0.270)	.05				0.145 (0.133)	.27	
Smoking										
Ex-smoking			0.149 (0.171)	.38				0.137 (0.084)	.10	
Current smoking			0.149 (0.229)	.52				0.176 (0.113)	.12	
Depression			-0.226 (0.181)	.21				-0.157 (0.089)	.08	
Alcohol			0.010 (0.007)	.16				0.004 (0.004)	.26	
Education										
10 years			-0.121 (0.264)	.65				-0.030 (0.129)	.82	
> 10 years			-0.209 (0.272)	.44				-0.123 (0.133)	.36	
Mental health			-0.012 (0.010)	.21				-0.007 (0.005)	.13	
Physical activity			0.131 (0.177)	.46				0.117 (0.087)	.18	

**(b) ODI regression models**										
	**WMH volume[a]**					**WMH count[a]**				
	**ODI**		**covariate**		**adjusted R^2**	**ODI**		**covariate**		**adjusted R^2**
	***β* (se)**	***p*-value**	***β* (se)**	***p*-value**		***β* (se)**	***p*-value**	***β* (se)**	***p*-value**	
**OSA model**	0.033 (0.009)	.001*			0.430	0.011 (0.005)	.02*			0.388
**Hypertension**	0.032 (0.010)	.001*	0.109 (0.170)	.52	0.429	0.010 (0.005)	.03*	0.075 (0.084)	.37	0.388
**Waist circumference**	0.033 (0.010)	.001*	-0.001 (0.007)	.92	0.429	0.011 (0.005)	.02*	-0.001 (0.004)	.73	0.387
**Triglycerides**	0.030 (0.009)	.002*	0.140 (0.070)	.05*	0.433	0.010 (0.005)	.04*	0.058 (0.035)	.10	0.390
**Total cholesterol**	0.032 (0.009)	.001*	0.072 (0.073)	.33	0.430	0.011 (0.005)	.02*	-0.030 (0.036)	.41	0.388
**LDL**	0.032 (0.009)	.001*	0.063 (0.085)	.46	0.430	0.011 (0.005)	.02*	-0.034 (0.042)	.42	0.388
**HDL**	0.033 (0.010)	.001*	-0.002 (0.244)	.99	0.429	0.011 (0.005)	.03*	-0.081 (0.120)	.50	0.387
**Diabetes mellitus**	0.030 (0.009)	.002*	0.543 (0.257)	.04*	0.434	0.010 (0.005)	.03*	0.179 (0.127)	.16	0.389
**Smoking**	0.032 (0.009)	.001*			0.430	0.010 (0.005)	.03*			0.392
Ex-smoking			0.187 (0.170)	.27				0.156 (0.083)	.06	
Current smoking			0.239 (0.220)	.28				0.203 (0.108)	.06	

© 2021 Zacharias HU et al. *JAMA Network Open.*

**Depression**	0.032 (0.009)	.001*	-0.117 (0.171)	.50	0.429	0.011 (0.005)	.02*	-0.087 (0.084)	.30	0.388
**Alcohol**	0.033 (0.009)	.001*	0.011 (0.007)	.12	0.432	0.011 (0.005)	.02*	0.005 (0.003)	.18	0.389
**Education**	0.032 (0.009)	.001*			0.430	0.011 (0.005)	.02*			0.388
10 years			-0.175 (0.261)	.51				-0.037 (0.129)	.78	
> 10 years			-0.328 (0.264)	.22				-0.142 (0.130)	.28	
**Mental health**	0.033 (0.009)	.001*	-0.010 (0.009)	.27	0.430	0.011 (0.005)	.02*	-0.007 (0.005)	.15	0.389
**Physical activity**	0.033 (0.009)	.001*	0.117 (0.174)	.50	0.429	0.012 (0.005)	.01*	0.117 (0.086)	.17	0.389
**Full model**	0.030 (0.010)	.003*			0.433	0.010 (0.005)	.04*			0.398
Hypertension			0.093 (0.180)	.61				0.070 (0.088)	.43	
Waist circumference			-0.009 (0.009)	.28				-0.006 (0.004)	.18	
Triglycerides			0.217 (0.118)	.07				0.119 (0.058)	.04*	
Total cholesterol			-0.280 (0.289)	.33				-0.212 (0.142)	.14	
LDL			0.351 (0.306)	.25				0.176 (0.150)	.24	
HDL			0.406 (0.428)	.34				0.174 (0.210)	.41	
Diabetes mellitus			0.509 (0.271)	.06				0.139 (0.133)	.30	
Smoking										
Ex-smoking			0.167 (0.171)	.33				0.142 (0.084)	.09	
Current smoking			0.160 (0.230)	.49				0.179 (0.113)	.11	
Depression			-0.216 (0.181)	.24				-0.154 (0.089)	.09	
Alcohol			0.009 (0.007)	.20				0.004 (0.004)	.30	
Education										
10 years			-0.105 (0.264)	.69				-0.025 (0.129)	.85	
> 10 years			-0.211 (0.272)	.44				-0.123 (0.133)	.36	
Mental health			-0.012 (0.010)	.22				-0.007 (0.005)	.13	
Physical activity			0.136 (0.177)	.44				0.119 (0.087)	.17	

© 2021 Zacharias HU et al. *JAMA Network Open.*

Supplementary Table S6. Results of the linear regression analysis for white matter hyperintensities (WMH) volume and count data with respect to categorized obstructive sleep apnea diagnostic criteria (a) apnea-hypopnea index (OSA_{AHI}) and (b) oxygen-desaturation index (OSA_{ODI}) after extending the respective OSA models by additional metabolic, vascular, and lifestyle covariates. All models were adjusted for sex, age modelled by a restricted cubic spline, intracranial volume, and body height and are based on the complete study sample of 529 SHIP-Trend-0 study participants. For each new covariates model, the estimated effect sizes (*β*-coefficients), standard errors (se), and *p*-values of both the respective OSA parameter and the considered additional covariate are given. No OSA was considered as the reference category. Abbreviations: AHI, apnea-hypopnea index; ICV, intracranial volume; HDL, high-density lipoprotein; LDL, low-density lipoprotein; ODI, oxygen desaturation index; OSA, obstructive sleep apnea; rcs, restricted cubic spline; se, standard error. **P*-value < 0.05; [a]WMH volume and count data have been log_2-transformed after the addition of a pseudocount of 1.

© 2021 Zacharias HU et al. *JAMA Network Open.*

(a) AHI regression models																		
	WMH volume[a]									WMH count[a]								
	AHI						covariate		adjusted R^2	AHI						covariate		adjusted R^2
	mild OSA_{AHI}		moderate OSA_{AHI}		severe OSA_{AHI}					mild OSA_{AHI}		moderate OSA_{AHI}		severe OSA_{AHI}				
	β (se)	*p*-value	*β* (se)	*p*-value	*β* (se)	*p*-value	*β* (se)	*p*-value		*β* (se)	*p*-value	*β* (se)	*p*-value	*β* (se)	*p*-value	*β* (se)	*p*-value	
**OSA model**	0.312 (0.197)	.12	0.480 (0.270)	.08	1.255 (0.341)	< .001*			0.431	0.129 (0.097)	.18	0.107 (0.133)	.42	0.419 (0.168)	.01*			0.387
**Hypertension**	0.305 (0.197)	.12	0.458 (0.272)	.09	1.232 (0.343)	< .001*	0.107 (0.170)	.53	0.430	0.125 (0.097)	.20	0.091 (0.134)	.50	0.402 (0.169)	.02*	0.078 (0.084)	.35	0.387
**Waist circumference**	0.321 (0.200)	.11	0.494 (0.276)	.07	1.278 (0.353)	< .001*	-0.002 (0.007)	.80	0.430	0.137 (0.099)	.17	0.119 (0.136)	.38	0.438 (0.174)	.01*	-0.002 (0.004)	.68	0.386
**Triglycerides**	0.270 (0.198)	.17	0.420 (0.271)	.12	1.166 (0.344)	.001*	0.130 (0.071)	.07	0.434	0.112 (0.098)	.25	0.082 (0.134)	.54	0.382 (0.170)	.03*	0.055 (0.035)	.12	0.389
**Total cholesterol**	0.313 (0.197)	.11	0.462 (0.270)	.09	1.249 (0.341)	< .001*	0.067 (0.073)	.37	0.431	0.129 (0.097)	.19	0.116 (0.133)	.39	0.422 (0.168)	.01*	-0.030 (0.036)	.40	0.387
**LDL**	0.309 (0.197)	.12	0.465 (0.271)	.09	1.244 (0.342)	< .001*	0.055 (0.086)	.52	0.430	0.131 (0.097)	.18	0.117 (0.134)	.38	0.427 (0.169)	.01*	-0.036 (0.042)	.40	0.387
**HDL**	0.319 (0.200)	.11	0.489 (0.273)	.07	1.265 (0.344)	< .001*	0.053 (0.247)	.83	0.430	0.120 (0.099)	.22	0.096 (0.135)	.48	0.407 (0.170)	.02*	-0.067 (0.122)	.59	0.386
**Diabetes mellitus**	0.265 (0.198)	.18	0.431 (0.270)	.11	1.193 (0.341)	.001*	0.528 (0.258)	.04*	0.435	0.114 (0.098)	.25	0.091 (0.133)	.50	0.399 (0.169)	.02*	0.174 (0.128)	.17	0.388
**Smoking**	0.298 (0.198)	.13	0.478 (0.270)	.08	1.219 (0.343)	< .001*			0.430	0.117 (0.097)	.23	0.105 (0.133)	.43	0.386 (0.169)	.02*			0.390
Ex-smoking							0.160 (0.171)	.35								0.146 (0.084)	.08	
Current smoking							0.227 (0.221)	.31								0.198 (0.109)	.07	
**Depression**	0.312 (0.197)	.11	0.479 (0.270)	.08	1.258 (0.341)	< .001*	-0.132 (0.171)	.44	0.431	0.130 (0.097)	.18	0.107 (0.133)	.42	0.421 (0.168)	.01*	-0.092 (0.084)	.28	0.387
**Alcohol**	0.312 (0.196)	.11	0.497 (0.269)	.07	1.273 (0.340)	< .001*	0.012 (0.007)	.09	0.433	0.129 (0.097)	.18	0.114 (0.133)	.39	0.426 (0.168)	.01*	0.005 (0.003)	.16	0.388
**Education**	0.294 (0.197)	.14	0.477 (0.270)	.08	1.236 (0.342)	< .001*			0.431	0.120 (0.097)	.22	0.104 (0.133)	.43	0.405 (0.169)	.02*			0.387
10 years							-0.188 (0.262)	.47								-0.038 (0.129)	.77	
> 10 years							-0.328 (0.265)	.22								-0.139 (0.131)	.29	
**Mental health**	0.305 (0.197)	.12	0.482 (0.269)	.08	1.257 (0.341)	< .001*	-0.010 (0.009)	.29	0.431	0.125 (0.097)	.20	0.109 (0.133)	.42	0.421 (0.168)	.01*	-0.006 (0.005)	.17	0.388
**Physical activity**	0.320 (0.197)	.11	0.487 (0.270)	.07	1.270 (0.342)	< .001*	0.114 (0.174)	.51	0.430	0.138 (0.097)	.16	0.115 (0.133)	.39	0.435 (0.169)	.01*	0.116 (0.086)	.18	0.388
**Full model**	0.255 (0.203)	.21	0.465 (0.279)	.10	1.170 (0.357)	.002*			0.434	0.101 (0.100)	.31	0.118 (0.137)	.39	0.373 (0.175)	.03*			0.396

© 2021 Zacharias HU et al. *JAMA Network Open.*

Hypertension							0.100 (0.180)	.58									0.074 (0.088)	.40	
Waist circumference							-0.010 (0.009)	.25									-0.006 (0.004)	.17	
Triglycerides							0.216 (0.118)	.07									0.118 (0.058)	.04*	
Total cholesterol							-0.296 (0.290)	.31									-0.215 (0.143)	.13	
LDL							0.360 (0.307)	.24									0.178 (0.151)	.24	
HDL							0.454 (0.431)	.29									0.189 (0.212)	.37	
Diabetes mellitus							0.493 (0.272)	.07									0.134 (0.133)	.32	
Smoking																			
Ex-smoking							0.141 (0.171)	.41									0.133 (0.084)	.12	
Current smoking							0.149 (0.230)	.52									0.176 (0.113)	.12	
Depression							-0.228 (0.181)	.21									-0.158 (0.089)	.08	
Alcohol							0.010 (0.007)	.16									0.004 (0.004)	.27	
Education																			
10 years							-0.121 (0.264)	.65									-0.028 (0.130)	.83	
> 10 years							-0.219 (0.272)	.42									-0.124 (0.134)	.36	
Mental health							-0.012 (0.010)	.22									-0.007 (0.005)	.14	
Physical activity							0.129 (0.177)	.47									0.117 (0.087)	.18	

(b) ODI regression models

	**WMH volume[a]**									**WMH count[a]**								
	**ODI**						**covariate**		**adjusted R^2**	**ODI**						**covariate**		**adjusted R^2**
	**mild OSA_{ODI}**		**moderate OSA_{ODI}**		**severe OSA_{ODI}**					**mild OSA_{ODI}**		**moderate OSA_{ODI}**		**severe OSA_{ODI}**				
	***β* (se)**	***p*-value**	***β* (se)**	***p*-value**	***β* (se)**	***p*-value**	***β* (se)**	***p*-value**		***β* (se)**	***p*-value**	***β* (se)**	***p*-value**	***β* (se)**	***p*-value**	***β* (se)**	***p*-value**	
**OSA model**	0.426 (0.237)	.07	1.030 (0.401)	.01*	1.130 (0.499)	.02*			0.429	0.141 (0.117)	.23	0.315 (0.197)	.11	0.538 (0.246)	.03*			0.388
**Hypertension**	0.408 (0.239)	.09	1.011 (0.402)	.01*	1.100 (0.501)	.03*	0.112 (0.170)	.51	0.428	0.129 (0.118)	.27	0.303 (0.198)	.13	0.519 (0.247)	.04*	0.073 (0.084)	.39	0.388

© 2021 Zacharias HU et al. *JAMA Network Open.*

**Waist circumference**	0.437 (0.243)	.07	1.048 (0.409)	.01*	1.153 (0.510)	.03*	-0.002 (0.008)	.83	0.428	0.154 (0.119)	.20	0.336 (0.201)	.10	0.565 (0.251)	.03*	-0.002 (0.004)	.60	0.387
**Triglycerides**	0.407 (0.237)	.09	0.974 (0.401)	.02*	0.999 (0.502)	.05*	0.141 (0.070)	.05*	0.432	0.134 (0.117)	.25	0.293 (0.198)	.14	0.487 (0.247)	.05	0.056 (0.035)	.11	0.390
**Total cholesterol**	0.422 (0.238)	.08	1.014 (0.401)	.01*	1.134 (0.499)	.02*	0.069 (0.073)	.35	0.428	0.143 (0.117)	.22	0.322 (0.198)	.10	0.536 (0.246)	.03*	-0.030 (0.036)	.40	0.388
**LDL**	0.420 (0.238)	.08	1.011 (0.402)	.01*	1.132 (0.499)	.02*	0.062 (0.086)	.47	0.428	0.145 (0.117)	.22	0.326 (0.198)	.10	0.537 (0.246)	.03*	-0.034 (0.042)	.43	0.388
**HDL**	0.425 (0.238)	.08	1.028 (0.402)	.01*	1.128 (0.500)	.03*	-0.020 (0.244)	.94	0.428	0.135 (0.117)	.25	0.305 (0.198)	.13	0.529 (0.246)	.03*	-0.085 (0.120)	.48	0.387
**Diabetes mellitus**	0.371 (0.238)	.12	1.027 (0.400)	.01*	0.952 (0.505)	.06	0.523 (0.261)	.05*	0.432	0.125 (0.118)	.29	0.314 (0.197)	.11	0.484 (0.249)	.05	0.159 (0.129)	.22	0.389
**Smoking**	0.412 (0.238)	.09	1.014 (0.402)	.01*	1.085 (0.500)	.03*			0.428	0.129 (0.117)	.27	0.301 (0.197)	.13	0.500 (0.246)	.04*			0.391
Ex-smoking							0.172 (0.171)	.32								0.150 (0.084)	.08	
Current smoking							0.233 (0.221)	.29								0.197 (0.108)	.07	
**Depression**	0.427 (0.238)	.07	1.016 (0.402)	.01*	1.132 (0.499)	.02*	-0.108 (0.171)	.53	0.428	0.142 (0.117)	.23	0.303 (0.198)	.13	0.540 (0.246)	.03*	-0.085 (0.084)	.31	0.388
**Alcohol**	0.416 (0.237)	.08	1.029 (0.401)	.01*	1.107 (0.499)	.03*	0.010 (0.007)	.14	0.430	0.137 (0.117)	.24	0.315 (0.197)	.11	0.529 (0.246)	.03*	0.004 (0.003)	.22	0.389
**Education**	0.407 (0.238)	.09	1.007 (0.404)	.01*	1.107 (0.502)	.03*			0.428	0.130 (0.117)	.27	0.309 (0.199)	.12	0.519 (0.247)	.04*			0.388
10 years							-0.134 (0.264)	.61								-0.031 (0.130)	.81	
> 10 years							-0.292 (0.266)	.27								-0.134 (0.131)	.31	
**Mental health**	0.402 (0.239)	.09	1.045 (0.401)	.01*	1.140 (0.499)	.02*	-0.010 (0.009)	.30	0.429	0.125 (0.117)	.29	0.325 (0.197)	.10	0.545 (0.245)	.03*	-0.006 (0.005)	.16	0.389
**Physical activity**	0.435 (0.238)	.07	1.051 (0.402)	.01*	1.151 (0.500)	.02*	0.124 (0.175)	.48	0.428	0.150 (0.117)	.20	0.335 (0.198)	.09	0.559 (0.246)	.02*	0.121 (0.086)	.16	0.389
**Full model**	0.348 (0.246)	.16	1.017 (0.414)	.02*	0.870 (0.521)	.10			0.431	0.114 (0.120)	.35	0.329 (0.203)	.11	0.435 (0.255)	.09			0.397
Hypertension							0.101 (0.180)	.58								0.072 (0.088)	.42	
Waist circumference							-0.010 (0.009)	.25								-0.006 (0.004)	.15	
Triglycerides							0.226 (0.119)	.06								0.118 (0.058)	.04*	
Total cholesterol							-0.299 (0.290)	.31								-0.211 (0.142)	.14	
LDL							0.367 (0.307)	.23								0.175 (0.150)	.24	

© 2021 Zacharias HU et al. *JAMA Network Open.*

HDL							0.407 (0.430)	.35								0.164 (0.211)	.44	
Diabetes mellitus							0.502 (0.274)	.07								0.127 (0.134)	.35	
Smoking																		
Ex-smoking							0.156 (0.171)	.36								0.137 (0.084)	.10	
Current smoking							0.158 (0.230)	.49								0.175 (0.113)	.12	
Depression							-0.201 (0.182)	.27								-0.150 (0.089)	.09	
Alcohol							0.009 (0.007)	.23								0.004 (0.004)	.32	
Education																		
10 years							-0.057 (0.267)	.83								-0.017 (0.131)	.90	
> 10 years							-0.176 (0.274)	.52								-0.117 (0.134)	.39	
Mental health							-0.011 (0.010)	.25								-0.007 (0.005)	.14	
Physical activity							0.139 (0.177)	.44								0.121 (0.087)	.16	

© 2021 Zacharias HU et al. *JAMA Network Open.*

Supplementary Table S7. Results of the linear regression analysis for white matter hyperintensity (WMH) volumes and counts with respect to an interaction term between obstructive sleep apnea diagnostic criteria and different metabolic, vascular, and lifestyle risk factors, which were tested separately in individual regression models. All models were adjusted for sex, age modelled by a restricted cubic spline, intracranial volume, and body height and are based on the complete study sample of 529 SHIP-Trend-0 study participants. Abbreviations: AHI, apnea-hypopnea index; HDL, high-density lipoprotein; ICV, intracranial volume; LDL, low-density lipoprotein; ODI, oxygen desaturation index; rcs, restricted cubic spline; se, standard error. **P*-value < 0.05; [a]WMH volume and count data have been $\log_2$-transformed after the addition of a pseudocount of 1.

© 2021 Zacharias HU et al. *JAMA Network Open.*

(a) AHI regression models

	WMH volume[a]							WMH count[a]						
	AHI		covariate		AHI*covariate		adjusted R^2	AHI		covariate		AHI*covariate		adjusted R^2
	β **(se)**	***p*-value**	***β*** **(se)**	***p*-value**	***β*** **(se)**	***p*-value**		***β*** **(se)**	***p*-value**	***β*** **(se)**	***p*-value**	***β*** **(se)**	***p*-value**	
**OSA model**	0.024 (0.006)	< .001*					0.432	0.008 (0.003)	.01*					0.389
**Hypertension**	0.026 (0.014)	.06	0.126 (0.202)	.53	-0.003 (0.015)	.83	0.430	0.009 (0.007)	.20	0.085 (0.099)	.40	-0.002 (0.008)	.83	0.387
**Waist circum-ference**	-0.033 (0.059)	.58	-0.005 (0.008)	.54	0.001 (0.001)	.33	0.431	-0.035 (0.029)	.23	-0.004 (0.004)	.30	0.0004 (0.0003)	.14	0.389
**Triglycerides**	0.020 (0.009)	.02*	0.111 (0.090)	.22	0.001 (0.003)	.76	0.433	0.004 (0.004)	.31	0.028 (0.044)	.52	0.001 (0.001)	.35	0.390
**Total choles-terol**	0.006 (0.033)	.86	0.038 (0.086)	.66	0.003 (0.006)	.58	0.431	0.009 (0.016)	.58	-0.032 (0.042)	.45	-0.0002 (0.003)	.95	0.387
**LDL**	0.009 (0.029)	.74	0.028 (0.100)	.78	0.004 (0.008)	.61	0.430	0.025 (0.014)	.08	-0.007 (0.049)	.90	-0.004 (0.004)	.23	0.389
**HDL**	0.038 (0.024)	.12	0.088 (0.267)	.74	-0.011 (0.018)	.55	0.430	0.007 (0.012)	.53	-0.074 (0.132)	.57	0.0001 (0.009)	.99	0.387
**Diabetes mellitus**	0.018 (0.007)	.01*	0.208 (0.336)	.54	0.027 (0.017)	.11	0.438	0.005 (0.003)	.15	-0.012 (0.166)	.94	0.015 (0.008)	.07	0.393
**Smoking**	0.025 (0.011)	.02*					0.430	0.005 (0.005)	.40					0.393
Ex-smoking			0.232 (0.202)	.25	-0.007 (0.014)	.63				0.156 (0.099)	.12	0.001 (0.007)	.93	
Current smoking			0.165 (0.251)	.51	0.009 (0.018)	.62				0.106 (0.123)	.39	0.014 (0.009)	.12	
**Lifetime Depression**	0.025 (0.007)	.001*	-0.100 (0.199)	.62	-0.004 (0.014)	.78	0.430	0.006 (0.004)	.09	-0.144 (0.098)	.14	0.007 (0.007)	.29	0.389
**Alcohol**	0.015 (0.009)	.10	0.004 (0.008)	.62	0.001 (0.001)	.10	0.436	0.005 (0.004)	.25	0.002 (0.004)	.56	0.0003 (0.0003)	.29	0.390
**Education**	0.042 (0.016)	.01*					0.431	0.012 (0.008)	.14					0.387
10 years			0.038 (0.324)	.91	-0.022 (0.018)	.24				0.025 (0.160)	.88	-0.007 (0.009)	.46	
> 10 years			-0.093 (0.329)	.78	-0.023 (0.020)	.26				-0.109 (0.162)	.50	-0.002 (0.010)	.85	
**Subjective Mental health**	0.009 (0.054)	.87	-0.012 (0.011)	.29	0.0003 (0.001)	.77	0.431	0.014 (0.026)	.61	-0.006 (0.006)	.30	-0.0001 (0.0005)	.83	0.389
**Physical activity**	0.039 (0.010)	< .001*	0.320 (0.205)	.12	-0.024 (0.013)	.06	0.434	0.013 (0.005)	.01*	0.179 (0.101)	.08	-0.007 (0.006)	.24	0.390

© 2021 Zacharias HU et al. *JAMA Network Open.*

**(b) ODI regression models**														
	**WMH volume[a]**							**WMH count[a]**						
	**ODI**		**covariate**		**ODI*covariate**		**adjusted R^2**	**ODI**		**covariate**		**ODI*covariate**		**adjusted R^2**
	**β (se)**	***p*-value**	**β (se)**	***p*-value**	**β (se)**	***p*-value**		**β (se)**	***p*-value**	**β (se)**	***p*-value**	**β (se)**	***p*-value**	
**OSA model**	0.033 (0.009)	.001*					0.430	0.011 (0.005)	.02*					0.388
**Hypertension**	0.043 (0.023)	.06	0.149 (0.185)	.42	-0.014 (0.025)	.58	0.429	0.017 (0.011)	.15	0.096 (0.091)	.29	-0.007 (0.012)	.55	0.387
**Waist circum-ference**	-0.027 (0.097)	.78	-0.002 (0.008)	.78	0.001 (0.001)	.54	0.428	-0.047 (0.048)	.33	-0.003 (0.004)	.48	0.001 (0.0005)	.22	0.388
**Triglycerides**	0.029 (0.013)	.03*	0.134 (0.085)	.12	0.001 (0.004)	.90	0.432	0.006 (0.006)	.36	0.035 (0.042)	.41	0.002 (0.002)	.33	0.390
**Total cholesterol**	0.004 (0.056)	.94	0.055 (0.081)	.50	0.005 (0.010)	.61	0.429	0.013 (0.028)	.64	-0.029 (0.040)	.47	-0.0003 (0.005)	.94	0.387
**LDL**	0.038 (0.049)	.44	0.067 (0.093)	.47	-0.001 (0.013)	.91	0.428	0.051 (0.024)	.03*	-0.003 (0.046)	.96	-0.011 (0.006)	.09	0.390
**HDL**	0.018 (0.037)	.62	-0.030 (0.254)	.91	0.012 (0.029)	.69	0.428	0.001 (0.018)	.94	-0.098 (0.125)	.43	0.007 (0.014)	.61	0.387
**Diabetes mellitus**	0.027 (0.010)	.01*	0.398 (0.306)	.20	0.020 (0.023)	.39	0.434	0.007 (0.005)	.16	0.066 (0.151)	.67	0.016 (0.012)	.17	0.390
**Smoking**	0.028 (0.016)	.08					0.428	0.002 (0.008)	.81					0.395
Ex-smoking			0.188 (0.186)	.31	0.001 (0.020)	.94				0.138 (0.091)	.13	0.007 (0.010)	.46	
Current smoking			0.178 (0.236)	.45	0.020 (0.027)	.47				0.110 (0.115)	.34	0.030 (0.013)	**.03***	
**Lifetime Depression**	0.031 (0.010)	.003*	-0.148 (0.186)	.43	0.010 (0.024)	.68	0.429	0.008 (0.005)	.13	-0.147 (0.092)	.11	0.019 (0.012)	.10	0.390
**Alcohol**	0.016 (0.013)	.21	0.004 (0.008)	.57	0.002 (0.001)	.07	0.434	0.006 (0.006)	.32	0.003 (0.004)	.47	0.0004 (0.0004)	.32	0.389
**Education**	0.056 (0.023)	.02*					0.429	0.016 (0.012)	.17					0.386
10 years			-0.032 (0.296)	.91	-0.025 (0.026)	.34				0.002 (0.146)	.99	-0.008 (0.013)	.56	
> 10 years			-0.160 (0.300)	.59	-0.034 (0.029)	.24				-0.117 (0.148)	.43	-0.004 (0.014)	.78	
**Subjective Mental health**	-0.052 (0.080)	.52	-0.015 (0.010)	.14	0.001 (0.001)	.29	0.430	0.012 (0.039)	.76	-0.006 (0.005)	.20	-0.00001 (0.001)	.98	0.388
**Physical activity**	0.053 (0.014)	< .001*	0.255 (0.190)	.18	-0.033 (0.018)	.07	0.432	0.017 (0.007)	.02*	0.156 (0.094)	.10	-0.009 (0.009)	.30	0.389

© 2021 Zacharias HU et al. *JAMA Network Open.*

Supplementary Table S8. Results of the linear regression analysis for periventricular frontal and periventricular dorsal white matter hyperintensity (WMH) volumes with respect to obstructive sleep apnea (OSA) diagnostic criteria (a) apnea-hypopnea index (AHI) and (b) oxygen-desaturation index (ODI) after extending the respective OSA models by additional metabolic, vascular, and lifestyle covariates. For each new covariates model, the estimated effect sizes (*β*-coefficients), standard errors (se), and *p*-values of both the respective OSA parameter and the considered additional covariate are given. All models were adjusted for sex, age modelled by a restricted cubic spline, intracranial volume, and body height and are based on a subsample of 392 SHIP-Trend-0 study participants. Abbreviations: AHI, apnea-hypopnea index; ICV, intracranial volume; HDL, high-density lipoprotein; LDL, low-density lipoprotein; ODI, oxygen desaturation index; OSA, obstructive sleep apnea; rcs, restricted cubic spline; se, standard error. **P*-value < 0.0125; [a]Regional WMH volumes have been log_2-transformed after the addition of a pseudocount of 1.

**(a) AHI regression models**										
	**Periventricular frontal WMH volume[a]**					**Periventricular dorsal WMH volume[a]**				
	**AHI**		**covariate**		**adjusted *R²***	**AHI**		**covariate**		**adjusted *R²***
	***β* (se)**	***p*-value**	***β* (se)**	***p*-value**		***β* (se)**	***p*-value**	***β* (se)**	***p*-value**	
**OSA model**	0.027 (0.007)	< .001*			0.417	0.016 (0.006)	.009*			0.196
**Hypertension**	0.027 (0.008)	< .001*	0.064 (0.173)	.71	0.416	0.016 (0.006)	.01*	0.167 (0.143)	.24	0.197
**Waist circumference**	0.027 (0.008)	< .001*	0.001 (0.008)	.93	0.415	0.016 (0.006)	.01	-0.0001 (0.006)	.99	0.194
**Triglycerides**	0.023 (0.008)	.003*	0.217 (0.069)	.002*	0.430	0.013 (0.006)	.03	0.143 (0.057)	.01	0.207
**Total cholesterol**	0.027 (0.007)	< .001*	0.186 (0.075)	.02	0.425	0.016 (0.006)	.01*	0.061 (0.063)	.33	0.196
**LDL**	0.027 (0.007)	< .001*	0.190 (0.088)	.03	0.423	0.016 (0.006)	.01*	0.036 (0.073)	.62	0.195
**HDL**	0.026 (0.008)	.001*	-0.323 (0.248)	.19	0.418	0.015 (0.006)	.02	-0.216 (0.205)	.29	0.197
**Diabetes mellitus**	0.024 (0.007)	.002*	1.000 (0.275)	< .001*	0.435	0.015 (0.006)	.02	0.448 (0.230)	.05	0.202
**Smoking**	0.027 (0.008)	< .001*			0.415	0.016 (0.006)	.01*			0.201
Ex-smoking			-0.113 (0.173)	.51				0.189 (0.142)	.19	
Current smoking			0.066 (0.224)	.77				0.354 (0.184)	.06	
**Depression**	0.027 (0.007)	< .001*	0.194 (0.175)	.27	0.417	0.016 (0.006)	.009*	0.150 (0.144)	.30	0.196
**Alcohol**	0.028 (0.007)	< .001*	0.005 (0.007)	.51	0.416	0.017 (0.006)	.007*	0.015 (0.006)	.01*	0.208
**Education**	0.026 (0.008)	.001*			0.422	0.016 (0.006)	.011*			0.193
10 years				.10					.58	

© 2021 Zacharias HU et al. *JAMA Network Open.*

> 10 years			-0.484 (0.288)<br>-0.648 (0.288)	.03*				-0.134 (0.240)<br>-0.180 (0.240)	.45	
**Mental health**	0.028 (0.007)	< .001*	-0.007 (0.009)	.44	0.416	0.017 (0.006)	.008*	-0.005 (0.008)	.52	0.195
**Physical activity**	0.028 (0.008)	< .001*	0.013 (0.182)	.94	0.415	0.015 (0.006)	.02	-0.233 (0.150)	.12	0.199
**Full model**	0.022 (0.008)	.005*			0.442	0.013 (0.006)	.05			
Hypertension			0.016 (0.180)	.93				0.135 (0.151)	.37	
Waist circumference			-0.016 (0.009)	.08				-0.012 (0.007)	.11	
Triglycerides			0.154 (0.120)	.20				0.069 (0.100)	.50	
Total cholesterol			0.079 (0.299)	.79				0.110 (0.250)	.66	
LDL			0.087 (0.314)	.78				-0.093 (0.263)	.72	
HDL			-0.292 (0.435)	.50				-0.298 (0.364)	.41	
Diabetes mellitus			0.965 (0.291)	.002*				0.467 (0.244)	.06	
Smoking										
Ex-smoking			-0.088 (0.171)	.61				0.189 (0.143)	.19	
Current smoking			-0.160 (0.233)	.49				0.151 (0.195)	.44	
Depression			0.138 (0.187)	.46				0.078 (0.157)	.62	
Alcohol			0.004 (0.007)	.59				0.012 (0.006)	.05	
Education										
10 years			-0.403 (0.288)	.16				-0.104 (0.241)	.67	
> 10 years			-0.523 (0.295)	.08				-0.135 (0.247)	.59	
Mental health			-0.004 (0.010)	.72				-0.003 (0.008)	.74	
Physical activity			0.014 (0.182)	.94				-0.211 (0.153)	.17	

(b) ODI regression models

	Periventricular frontal WMH volume[a]				
	ODI		covariate		adjusted R^2
	β **(se)**	***p*-value**	***β*** **(se)**	***p*-value**	
**OSA model**	0.038 (0.011)	.001*			0.414
**Hypertension**	0.038 (0.011)	.001*	0.057 (0.174)	.74	0.413
**Waist circumference**	0.038 (0.012)	.002*	0.001 (0.008)	.85	0.413
**Triglycerides**	0.033 (0.011)	.004*	0.229 (0.069)	.001*	0.429
**Total cholesterol**	0.038 (0.011)	.001*	0.192 (0.076)	.01*	0.422
**LDL**	0.037 (0.011)	.002*	0.195 (0.088)	.03	0.420
**HDL**	0.036 (0.011)	.002*	-0.348 (0.248)	.16	0.416
**Diabetes mellitus**	0.032 (0.011)	.005*	0.997 (0.277)	< .001*	0.432
**Smoking**	0.038 (0.011)	.001*			0.412
Ex-smoking			-0.080 (0.173)	.64	
				.68	

© 2021 Zacharias HU et al. *JAMA Network Open.*

Current smoking			0.093 (0.224)			
**Depression**	0.038 (0.011)	.001*	0.210 (0.175)	.23	0.415	
**Alcohol**	0.038 (0.011)	.001*	0.004 (0.007)	.59	0.413	
**Education**	0.036 (0.011)	.002*			0.419	
10 years			-0.462 (0.289)	.11		
> 10 years			-0.646 (0.289)	.03		
**Mental health**	0.038 (0.011)	.001*	-0.007 (0.009)	.44	0.414	
**Physical activity**	0.038 (0.011)	.001*	0.015 (0.182)	.94	0.413	
**Full model**	0.030 (0.012)	.01*			0.440	
Hypertension			0.005 (0.180)	.98		
Waist circumference			-0.015 (0.009)	.08		
Triglycerides			0.166 (0.120)	.17		
Total cholesterol			0.077 (0.299)	.80		
LDL			0.089 (0.315)	.78		
HDL			-0.293 (0.435)	.50		
Diabetes mellitus			0.949 (0.292)	.002*		
Smoking						
Ex-smoking			-0.063 (0.171)	.71		
Current smoking			-0.142 (0.233)	.54		
Depression			0.153 (0.187)	.42		
Alcohol			0.003 (0.007)	.67		
Education						
10 years			-0.389 (0.289)	.18		
> 10 years			-0.525 (0.295)	.08		
Mental health			-0.003 (0.010)	.76		
Physical activity			0.019 (0.183)	.92		

© 2021 Zacharias HU et al. *JAMA Network Open.*
